# The microbiota-gut-brain-axis theory: role of gut microbiota modulators (GMMs) in gastrointestinal, neurological, and mental health disorders

**DOI:** 10.1007/s00210-025-04155-2

**Published:** 2025-05-05

**Authors:** Al-Hassan Soliman Wadan, Mostafa K. Abd El-Aziz, Doha El-Sayed Ellakwa

**Affiliations:** 1 Oral Biology Department, Faculty of Dentistry, Galala University (15888), Galala Plateau, Attaka, Suez Governorate, Egypt; 2 Faculty of Pharmacy, King Salman International University, Ras-Sudr, South Sinai Egypt; 3 Clinical Pharmacology and Pharmacogenomics Research Group (CPPRG), Faculty of Pharmacy and Biotechnology, German University in Cairo-GUC, Cairo, Egypt; 4 Department of Biochemistry and Molecular Biology, Faculty of Pharmacy for Girls, Al-Azhar University, Cairo, Egypt; 5 Department of Biochemistry, Faculty of Pharmacy, Sinai University, Kantra Branch, Ismailia, Egypt

**Keywords:** Gut microbiota modulators, Prebiotics, Probiotics, Microbiota-gut-brain axis, Fecal transplantation (FMT), Drug repurposing

## Abstract

The modulation of gut microbiota presents promising therapeutic possibilities for various health conditions, ranging from gastrointestinal infections to neurodegenerative and mental health disorders. Among the available interventions, gut microbiota modulators (GMMs) such as probiotics and prebiotics have demonstrated significant potential in infection prevention and neuroprotection. Despite these encouraging findings, the clinical application of GMMs remains challenging due to safety concerns and inconsistent effectiveness across diverse patient populations. These factors create substantial barriers to the widespread adoption of microbiota-based therapies in clinical practice. To overcome these challenges and fully leverage the therapeutic potential of microbiota modulation, this review explores the feasibility of repurposing GMMs for managing multiple health disorders. A broad spectrum of microbiota-targeted strategies is examined, including dietary modifications, fecal microbiota transplantation, bacteriophage therapy, microbiome engineering, and immune system modulation. A particularly innovative approach involves integrating GMMs with pharmaceutical delivery systems to enhance therapeutic efficacy while mitigating potential adverse effects. This integrative strategy underscores the pivotal role of the gut microbiome in health and disease, supporting the development of precision medicine tailored to individual patient needs. By combining GMMs with targeted delivery mechanisms, this approach not only improves treatment effectiveness but also addresses critical concerns regarding safety and patient variability. Furthermore, this review outlines future research directions within the rapidly evolving field of microbiota modulation, emphasizing the necessity of comprehensive clinical trials and long-term safety evaluations. By critically assessing both the challenges and opportunities associated with microbiota-based interventions, this study provides a strategic framework for translating experimental research into viable clinical applications. A holistic approach to gut microbiota modulation has the potential to redefine treatment paradigms, offering personalized therapeutic strategies for a wide range of disorders and advancing the broader field of precision medicine.

## Introduction

Microbiota modulation, defined as the deliberate modification of microbial populations, presents significant potential for addressing a multitude of health-related concerns (Charitos et al. 2024; Khan et al. 2023). Gut microbiota modulators (GMMs), encompassing agents such as probiotics and prebiotics, have demonstrated efficacy in the prophylaxis of gastrointestinal infections, including those caused by *Salmonella* and *Clostridium difficile*, as well as in delivering neuroprotective benefits pertinent to neurodegenerative conditions such as Alzheimer’s and Parkinson’s diseases (Ellakwa et al. 2025a; Park 2019). Furthermore, burgeoning evidence suggests their advantageous influence on mental health disorders, notably autism spectrum disorder and attention-deficit/hyperactivity disorder (Ellakwa et al. 2025; Pola et al. 2023). However, notwithstanding these encouraging outcomes, the translation of research findings into clinical applications poses considerable challenges, particularly regarding safety and effectiveness across heterogeneous patient populations (Ali et al. 2018; Papapetropoulos and Szabo 2018). Despite rapid advancements in science and technology, the development of synthetic drugs remains expensive and time-consuming, particularly in de novo drug development (Madkour et al. 2024; Ali et al. 2023). This underscores the need for alternatives like drug repurposing, which involves exploring new uses for already approved or previously ineffective drugs and can significantly reduce development costs and time (Aubé 2012; Lloyd-Price et al. 2016). Drug repurposing has recently gained popularity for giving existing medications new applications, such as repurposing *thalidomide* for erythema nodosum leprosum and multiple myeloma (Shaaban et al. 2022; Patrignani et al. 2024). The repurposing of existing drugs for gut microbiota modulation offers a substantial advantage over traditional de novo drug development, particularly in terms of cost-effectiveness and time efficiency (Shaaban et al. 2022; Patrignani et al. 2024). Conventional drug discovery is a highly resource-intensive process, often requiring more than a decade and incurring costs that can reach several billion dollars (Ellakwa et al. 2024a; Ashburn and Thor 2004). Furthermore, the high failure rates associated with novel molecular entities (NMEs) contribute to the inefficiency of this approach (Haripriya et al. 2024; Ellakwa et al. 2024b). In contrast, drug repurposing capitalizes on previously approved drugs that have already undergone extensive safety assessments, thereby significantly reducing the time required for early-stage research and development by an estimated 6 to 7 years (Dey 2019; Amoroso et al. 2020). Beyond accelerating the development timeline, drug repurposing also lowers overall research expenditures and increases the probability of clinical success (Ellakwa et al. 2024c; Poduri et al. 2023). Since repurposed drugs possess well-characterized pharmacological properties, they can be efficiently redirected toward treating conditions such as inflammatory bowel disease (IBD) with greater confidence in their safety and efficacy (Ellakwa and Ellakwa 2021; Amin et al. 2013). Given these advantages, drug repurposing presents a practical and efficient strategy for identifying and implementing gut microbiota modulators, offering a streamlined pathway for translating microbiome-targeted therapies into clinical practice (Ranjan 2022).

This review seeks to thoroughly assess the possibility of drug repurposing for modulating gut microbiota, concentrating on the identification of interesting candidates from established pharmacological libraries. During this review, we want to accelerate the development of novel GMMs by utilizing modern screening methods and current safety data, thereby reducing the risks and expenses linked to conventional drug discovery. This method could profoundly affect microbiome regulation, providing novel therapeutic alternatives for many health issues influenced by gut microbiota. Moreover, we examine the incorporation of GMMs into pharmaceutical delivery mechanisms as a novel methodology to augment therapeutic effectiveness while minimizing adverse reactions. This approach underscores the critical role of the gut microbiome in both health and pathology, thereby presenting a trajectory toward precision medicine that is customized to individual patient requirements. The review further articulates potential avenues for future investigative endeavors within this swiftly advancing domain, accentuating the necessity for comprehensive clinical trials and prolonged safety evaluations. By confronting the challenges and prospects inherent in microbiota modulation, we aspire to expedite the progression of safe and efficacious treatments that leverage the capabilities of the gut microbiome to enhance outcomes across a spectrum of disorders. This review will focus on the recent advances regarding the use of GMMs for the treatment of gastrointestinal infections, neurodegenerative disorders, and mental health conditions.

## Gut microbiota: friends with benefits

Humans have undergone co-evolution alongside the vast and diverse microbial communities that reside within the body, forming intricate and highly specialized ecosystems adapted to specific bodily habitats. These microbial ecosystems continuously adjust to dynamic physiological changes within the host, maintaining a delicate balance that supports overall health. Disruptions to this balance, known as dysbiosis, have been implicated in the development of various diseases. Research has linked microbiome dysbiosis to a range of conditions, including IBD, multiple sclerosis, type 1 and type 2 diabetes, allergies, asthma, autism, and certain forms of cancer. These associations highlight the crucial role of the microbiome in immune regulation, metabolic processes, and overall physiological homeostasis (Ranjan 2022).

**Fig. 1:**
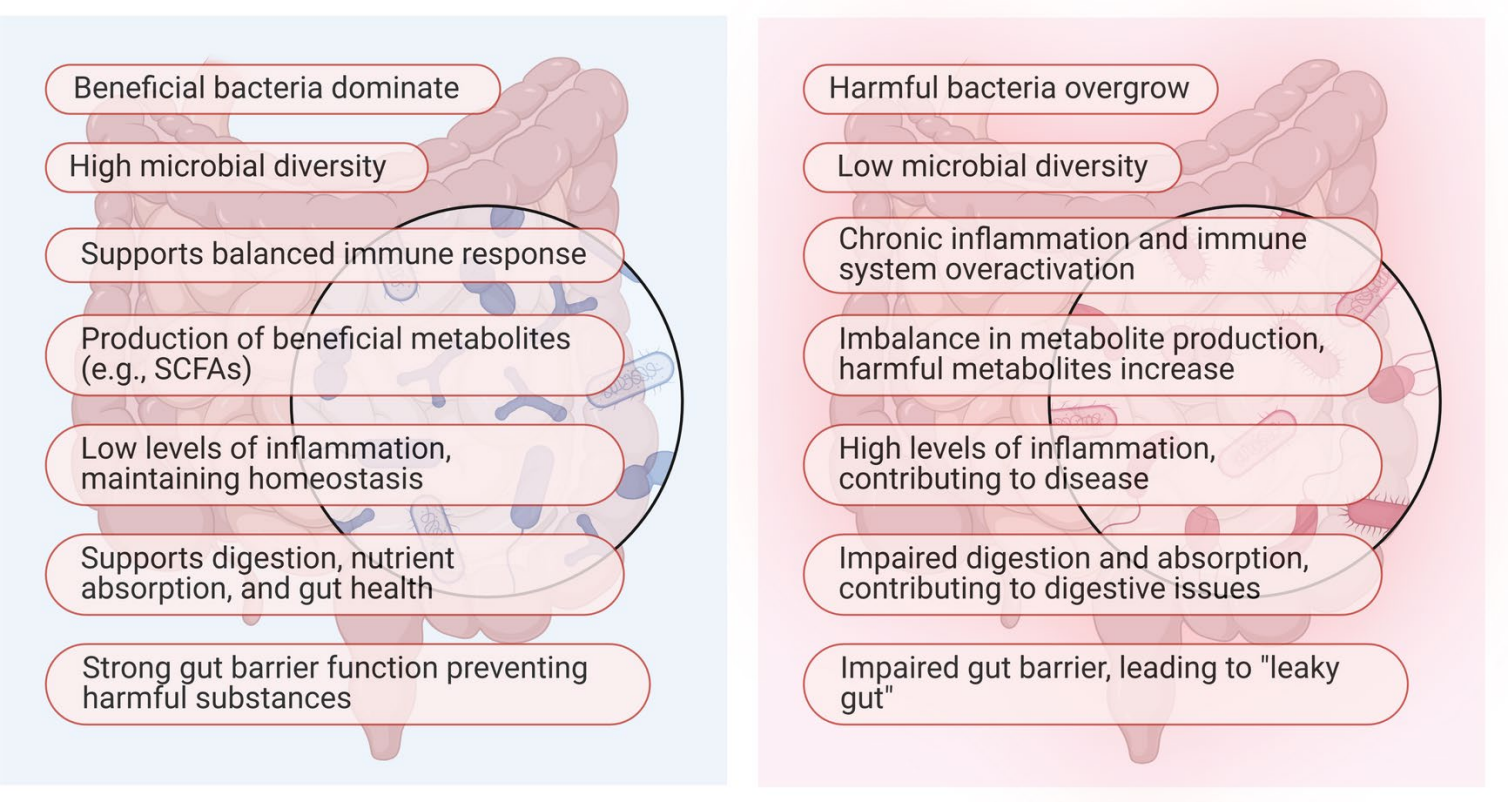
Comparison between a healthy versus a diseased gut microbiota, highlighting key characteristics

The human gut microbiota (GM) consists of trillions of microorganisms co-evolved with the human host (Ranjan 2022). These microorganisms produce a wide range of specific substances that influence the host, including lipids, glycolipids, oligosaccharides, amino acids, and non-ribosomal peptides, which possess immunomodulatory, cytotoxic, antioxidant, and antibacterial properties (Sicard et al. 2017). These substances play critical roles in immune modulation, metabolism, and neurological signaling. Hence, changes in the composition of the GM are directly related to multiple host diseases (Wadan et al. 2024). The GM can be classified into three categories based on their effects on host health. The beneficial bacteria promote health; conditionally, pathogenic bacteria remain harmless under normal conditions but may cause disease when the host’s immune system is compromised, and pathogenic bacteria are inherently harmful and contribute to disease (Fig. 1) (Haselbeck et al. 2017). The beneficial bacteria are the dominant type in the intestinal tract, playing a key role in maintaining intestinal homeostasis and modulating the immune system (Raza et al. 2024a). The metabolism of these beneficial bacteria yields several valuable materials for the host, leading to the emergence of substances like probiotics and prebiotics as promising domains in repurposing therapeutic modalities and treating various diseases, including gastrointestinal diseases, metabolic and cardiovascular diseases, neurodegenerative disorders, and immune and inflammatory diseases (Raza et al. 2024b). Additionally, GM can be repurposed to generate antibiotic compounds produced by bacteria isolated from the gut environment (Neamah et al. 2024).

A healthy gut microbiota is distinguished by a diverse and balanced microbial community consisting of bacteria, viruses, fungi, and archaea that coexist in a symbiotic manner to support overall health. This microbial diversity plays a fundamental role in preserving gut barrier integrity, modulating immune responses, and optimizing digestion and nutrient absorption. In a healthy gut environment, beneficial bacterial species such as *Bifidobacterium* and *Lactobacillus* predominate, contributing to the production of short-chain fatty acids (SCFAs), including butyrate, acetate, and propionate (Oneto and Khanna 2024). These SCFAs serve as an energy source for gut epithelial cells, possess anti-inflammatory properties, and help sustain a slightly acidic intestinal environment that inhibits the proliferation of pathogenic microorganisms. Furthermore, a balanced gut microbiota facilitates gut-brain communication through the synthesis of neurotransmitters like serotonin and gamma-aminobutyric acid (GABA), both of which are essential for mental health and cognitive function. In contrast, a diseased gut microbiota, commonly referred to as dysbiosis, is characterized by a decline in microbial diversity and a disruption in the equilibrium between beneficial and harmful microorganisms (Ellakwa and Amin 2022). Dysbiosis is often marked by a reduction in protective bacterial species alongside an overgrowth of pathogenic microbes such as *Clostridium difficile*, *Escherichia coli*, and *Salmonella*. This microbial imbalance compromises gut barrier function, leading to increased intestinal permeability, commonly known as “leaky gut.” Consequently, harmful substances, including toxins and undigested food particles, can translocate into the bloodstream, provoking systemic inflammation and immune dysregulation. Additionally, dysbiosis is associated with diminished SCFA production, further impairing gut health and increasing susceptibility to infections and chronic diseases. The impact of dysbiosis extends beyond the gastrointestinal system, influencing overall physiological health and contributing to various disorders (Aron-Wisnewsky et al. 2020). An imbalanced gut microbiota has been strongly linked to gastrointestinal conditions such as irritable bowel syndrome (IBS), IBD, and colorectal cancer. Furthermore, dysbiosis is implicated in metabolic disorders, including obesity and type 2 diabetes, due to its role in altering energy metabolism and promoting insulin resistance. Disruptions in the gut-brain axis caused by dysbiosis are also associated with neurological and mental health disorders, including anxiety, depression, and neurodegenerative diseases such as Alzheimer’s and Parkinson’s disease. Pathogenic bacteria in a dysbiotic gut produce inflammatory molecules and toxins capable of crossing the blood–brain barrier, further exacerbating these conditions. A key distinction between healthy and diseased gut microbiota lies in their functional capabilities. A balanced microbiota efficiently degrades complex carbohydrates, synthesizes essential vitamins such as B vitamins and vitamin K, and aids in the detoxification of harmful substances. Conversely, a diseased gut microbiota exhibits impaired metabolic efficiency and an increased capacity for generating toxic byproducts, including ammonia, hydrogen sulfide, and endotoxins like lipopolysaccharides (LPS). These harmful metabolites contribute to chronic inflammation, oxidative stress, and tissue damage, creating a self-perpetuating cycle of disease progression (Ellakwa et al. 2021).

Furthermore, the microbiota–gut–brain axis (MGBA), a bidirectional communication network between the gut and the brain, is linked to various neurological disorders, including depression, anxiety, and neurodegenerative diseases like Parkinson’s (PD) and Alzheimer’s (AD). At the same time, GM dysbiosis is also strongly associated with metabolic disorders such as obesity, diabetes, and non-alcoholic fatty liver disease (Cani and Everard 2016). The aforementioned properties of GM heavily inspired the concept of modifying its components to yield specific substances or to affect intracellular pathways (McCabe and Parameswaran 2018). This modification process is carried out by GMMs, which are diverse and versatile (Table 1), which are substances or interventions that influence the composition and activity of the GM to maintain or restore a healthy balance of microorganisms within the gut, essential for various aspects of health, including digestion, immune function, and disease prevention (Garcia-Gutierrez et al. 2019). With the increasing knowledge of how gut microbiome contributes to GIT disorders and affects treatment outcomes, GMMs, aiming to restore gut microbial homeostasis, become a potential strategy for preventing and treating such conditions.

**Table 1:** This table summarizes strategies of gut microbiota modulation, including probiotics, prebiotics, postbiotics, antibiotics, FMT, and their putative mechanisms of action

GMM	Function
Probiotics	When consumed in adequate amounts (typically ranging from 10⁶ to 10^12^ CFU per day, depending on the strain and formulation), live beneficial bacteria that are beneficial to the host can help restore the balance of GM after disturbances, such as those caused by antibiotic use or gastrointestinal infections
Prebiotics	Non-digestible food components that promote the growth and activity of beneficial GM, with common examples including dietary fibers like inulin
Synbiotics	Formulations combine probiotics and prebiotics to enhance the survival and colonization of beneficial GM
Antibiotics	While antibiotics are commonly used to combat harmful bacteria, they can disrupt the balance of GM; however, their selective use may serve as a modulator by targeting specific pathogens
FMT	FMT involves transferring stool from a healthy donor to a patient to restore a balanced and diverse gut microbiota. It primarily treats conditions like Clostridium difficile infection by re-establishing healthy microbial communities and improving gut health

Prebiotics and probiotics are essential components of GMMs that contribute significantly to gastrointestinal, neurological, and mental health. Prebiotics refer to non-digestible dietary components, such as specific fibers, that selectively promote the growth and metabolic activity of beneficial gut bacteria. By serving as a substrate for these microorganisms, prebiotics facilitate their proliferation and enhance the production of SCFAs, including butyrate, acetate, and propionate (Ellakwa et al. 2024a). These metabolites play a crucial role in maintaining gut barrier integrity, mitigating inflammatory responses, and modulating immune function. Furthermore, prebiotics support brain health through the gut-brain axis, a bidirectional communication network connecting the gut microbiota with the CNS. By fostering a healthy gut environment, prebiotics may indirectly influence cognitive function and mental well-being (Ellakwa et al. 2024a). Conversely, probiotics are live microorganisms that, when administered in appropriate quantities, confer health benefits to the host. Strains such as *Lactobacillus* and *Bifidobacterium* colonize the gut, where they compete with pathogenic bacteria, thereby restoring microbial homeostasis. In addition to reinforcing the intestinal barrier by strengthening tight junctions between epithelial cells, probiotics produce antimicrobial compounds that inhibit the growth of harmful bacteria. Moreover, they interact with gut-associated lymphoid tissue (GALT), modulating immune responses and reducing systemic inflammation. Recent research suggests that probiotics may also influence neurological and mental health by synthesizing neurotransmitters such as serotonin and gamma-aminobutyric acid (GABA), both of which play critical roles in mood regulation and cognitive processes. The gut-brain axis serves as a fundamental pathway through which prebiotics and probiotics exert their effects on neurological and mental health disorders (Zipperer et al. 2016). This interaction is mediated by mechanisms such as vagus nerve signaling, immune modulation, and the production of microbial metabolites, including SCFAs and neurotransmitters. Dysbiosis, or an imbalance in gut microbiota composition, has been associated with a range of conditions, including anxiety, depression, and neurodegenerative diseases such as Alzheimer’s and Parkinson’s disease. By restoring microbial equilibrium, GMMs have the potential to mitigate symptoms of these disorders. Both prebiotics and probiotics have been shown to attenuate systemic inflammation, which is frequently elevated in neurological and mental health conditions, and to enhance levels of BDNF, a protein essential for neuronal growth and synaptic plasticity. Clinical evidence highlights the therapeutic potential of prebiotics and probiotics in the management of gastrointestinal disorders such as IBS, IBD, and antibiotic-associated diarrhea. In the context of neurological and mental health, probiotics have demonstrated efficacy in alleviating symptoms of depression and anxiety. In contrast, prebiotics have been linked to enhanced stress resilience and cognitive function. However, the effectiveness of GMMs is influenced by factors such as the specific strains utilized, dosage, and inter-individual differences in gut microbiota composition. As a result, personalized approaches to prebiotic and probiotic supplementation are essential to optimize their therapeutic potential (Ellakwa et al. 2024 d).

## Repurposing GMMs for gastrointestinal disease treatment

**Fig. 2:**
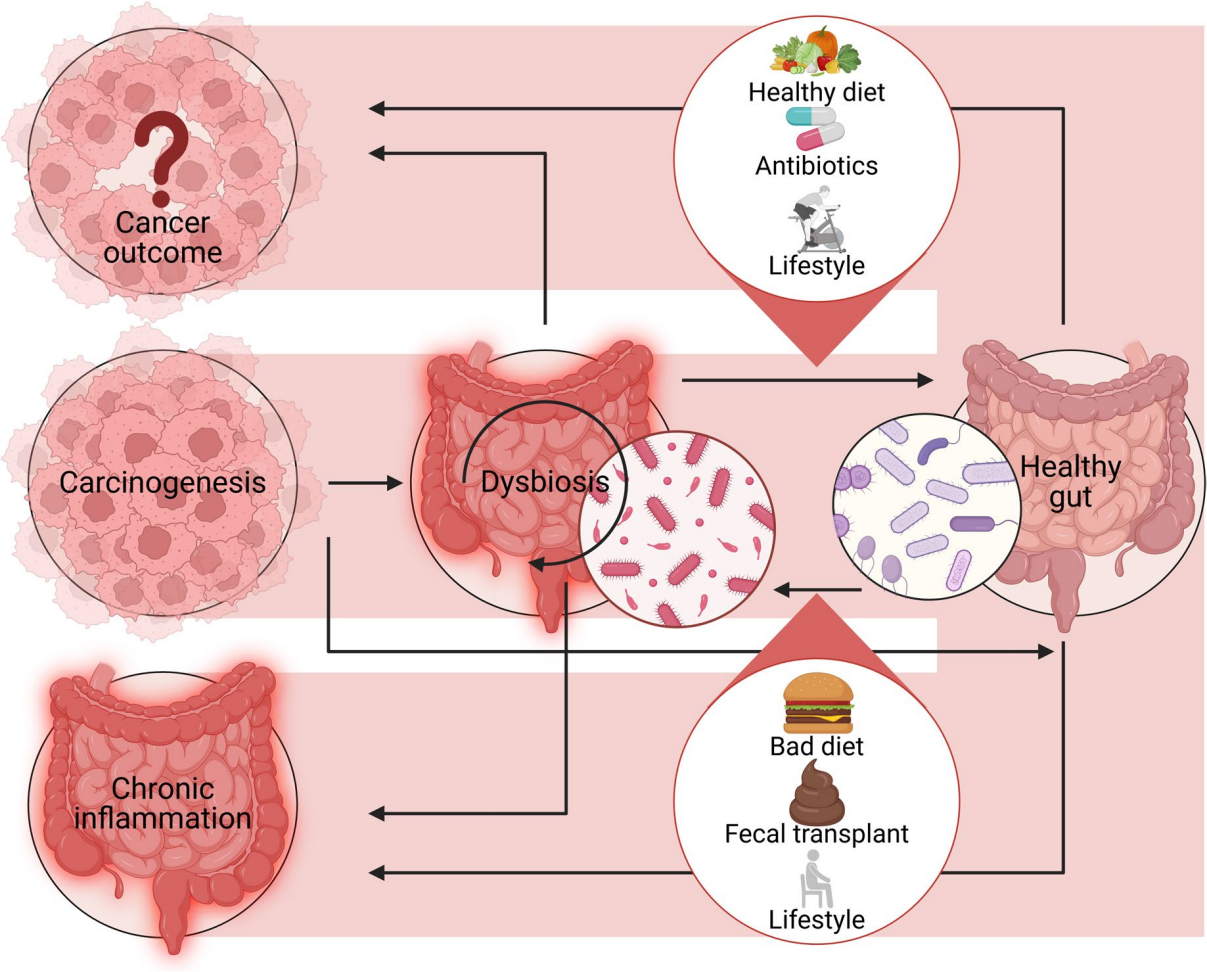
This schematic diagram depicts the impact of gut microbiota modulation on cancer progression. The figure illustrates how lifestyle factors such as diet and antibiotic use can shape the gut microbiome, potentially leading to a healthy gut or dysbiosis. Dysbiosis, driven by factors like an unhealthy diet and fecal transplants, contributes to chronic inflammation, a precursor to carcinogenesis, which ultimately influences cancer outcomes. In contrast, a healthy diet, balanced lifestyle, and the appropriate use of antibiotics support a healthy gut microbiota, reducing inflammation and mitigating cancer progression

GIT diseases refer to disorders affecting the gastrointestinal tract and its associated organs involved in digestion. This includes conditions that impact the esophagus, stomach, small and large intestines, and sometimes accessory organs such as the liver, pancreas, and gallbladder (Fig. 2) (Table 2). These disorders range from inflammatory and infectious diseases to functional and neoplastic conditions. Common examples include gastroesophageal reflux disease (GERD), irritable bowel syndrome (IBS), inflammatory bowel disease (IBD), peptic ulcer disease, and colorectal cancer. *Salmonella typhimurium* (*ST*), the most common serovar causing invasive nontyphoidal Salmonella infections, is a major contributor to gastroenteritis and is often linked to non-bloody diarrhea due to its complex interactions with the GM (Sharkey et al. 2018). Studies have shown that ST enhances its ability to colonize the gut by collaborating with beneficial microbiota that break down mucins (Ellakwa et al. 2024e). Current probiotics can prevent ST infections by directly interacting with pathogens, enhancing immune responses, competing for adhesion sites on the intestinal epithelium, and promoting mucin production to inhibit ST entry (Table 3) (Guery et al. 2019). Other probiotics, such as strains of *Lactobacillus* and *Bacillus*, induce immunomodulatory effects, including increased levels of IgA and cytokines, reduced bacterial dissemination, and enhanced anti-inflammatory activity (Samarkos et al. 2018).

**Table 2:** Comparison of GMMs and their efficacy in treating gut disorders. *IBD *inflammatory bowel disease, *IBS* irritable bowel syndrome, *FMT* fecal microbiota transplant

Disease	GMM type	Efficacy	Mechanism	Side effects
IBD	Probiotics (e.g., *Saccharomyces boulardii*)	Moderate to high	Immunomodulation, gut barrier enhancement	Bloating, mild gas
IBS	Prebiotics (e.g., *Fructooligosaccharides*)	Moderate	Promotion of beneficial gut flora	Abdominal discomfort
Antibiotic-associated diarrhea	Synbiotics (e.g., *Lactobacillus* with FOS)	High	Replenishing microbiota after antibiotic use	Rare: mild digestive upset
*Clostridium* difficile Infection	FMT	Very high	Restoration of gut microbiota diversity	Infection risk from donor
Metabolic disorders (obesity, diabetes)	SCFAs (e.g., Butyrate)	Moderate	Anti-inflammatory, insulin sensitivity	Mild gastrointestinal discomfort

**Table 3:** Probiotic strains that have been shown to prevent *Salmonella typhimurium* infections

Probiotic strain	Mechanism of action
*Escherichia coli* Nissle 1917	Competes with *S. typhimurium* for iron, reducing colonization
*Lactobacillus casei* CRL 431	Prevents and treats *Salmonella* infections in mouse models
*Lactobacillus plantarum* GX17	Reduces *Salmonella* counts in organs, alleviates infection-induced weight loss
*Lactobacillus acidophilus* 1.3251 and *Lactobacillus plantarum* 9513	Probiotic mixture shows therapeutic effects against *S. Typhimurium*

Moreover, *Clostridium difficile* infection (CDI) is a major cause of hospital-associated diarrhea, with recurrence rates of 20 to 30% (Dey and Ray Chaudhuri 2023). CDI is often linked to imbalances in GM, particularly in elderly individuals and those undergoing antibiotics, which can disrupt the microbial ecosystem (Ellakwa et al. 2024f). For instance, prolonged usage of antibiotics lowers the levels of short-chain fatty acids (SCFAs) that naturally limit the capacity of *C. difficile* to cause infection (Ellakwa and Ellakwa 2021; Amin et al. 2013). Resistance against CDI is established through several mechanisms: directly inhibiting CDI by increasing the production of SCFAs, competing for available nutrients, or indirectly stimulating immune defense networks (Shukla et al. 2024). Currently, the probiotic butyrate can increase SCFA levels, modulate immune responses, alter hypoxia-inducible factor pathways, and influence bile acid metabolism, collectively contributing to the inhibition of CDI (Zhong et al. 2025).

Repurposing GMMs for treating ST and CDI involves adapting treatments initially developed for other gut-related conditions to provide an alternative to traditional antibiotics, often exacerbating gut dysbiosis (Ellakwa et al. 2017). For instance, probiotics like *Lactobacillus* and *Bifidobacterium*, commonly used for dysbiosis and irritable bowel syndrome (IBS), can be repurposed to enhance intestinal barrier function, inhibit pathogen adhesion, and modulate immune responses in ST and CDI (Schroeder et al. 2018). Also, *Saccharomyces boulardii*, a probiotic known to prevent CDI recurrence, could be repurposed for ST infections, particularly for boosting mucosal immunity and reducing inflammation in patients with pre-existing gut disorders (Salem et al. 2019). Likewise, prebiotics such as inulin and fructooligosaccharides (FOS), currently used to promote general gut health and immune function, could enhance probiotic efficacy against these infections by inducing beneficial bacteria growth such as Bifidobacterium and Lactobacillus inhibiting pathogen colonization and restore microbial diversity after antibiotic treatment (Abdel-Hamid et al. 2018).

GMMs, including prebiotics, probiotics, and FMT, have gained recognition as effective therapeutic approaches for infections caused by *Clostridium difficile* and *Salmonella Typhimurium*. The administration and dosage of these interventions vary based on the type of treatment, the severity of the infection, and the overall health status of the patient. In the case of *C. difficile* infection (CDI), probiotics such as *Saccharomyces boulardii* and specific strains of *Lactobacillus* and *Bifidobacterium* are frequently utilized. The recommended dosage for *S. boulardii* typically ranges from 250 to 500 mg, taken orally twice daily. At the same time, probiotic formulations containing *Lactobacillus* and *Bifidobacterium* strains are often administered at doses of 10–50 billion CFUs per day. These probiotics are generally used in conjunction with antibiotic therapy to help reestablish gut microbial balance and minimize the risk of CDI recurrence. For cases of recurrent or severe CDI, FMT has demonstrated high efficacy as a treatment option. This procedure involves the transfer of processed stool from a healthy donor into the patient’s gastrointestinal tract via colonoscopy, nasogastric tube, or oral capsules. While FMT dosage is not standardized, it typically involves the administration of 30–50 g of donor stool, either suspended in a saline solution or encapsulated in freeze-dried form. Clinical studies have reported success rates exceeding 90% in resolving recurrent CDI, as FMT effectively restores microbial diversity and intestinal homeostasis (Cristofori et al. 2021). However, this intervention is generally reserved for patients who do not respond to conventional therapies, including antibiotics and probiotics. Infections caused by *Salmonella typhimurium* may also be managed with probiotics, which help alleviate symptoms and facilitate recovery. Commonly used strains, such as *Lactobacillus rhamnosus* GG and *Bifidobacterium lactis*, are administered in doses ranging from 10 to 20 billion CFUs per day. These probiotics inhibit *Salmonella* by competing for nutrients and adhesion sites in the gut, strengthening the intestinal barrier and modulating immune responses. Additionally, prebiotics such as fructooligosaccharides (FOS) and galactooligosaccharides (GOS) may be co-administered with probiotics to enhance the growth and activity of beneficial gut bacteria. Prebiotic dosages typically range from 5 to 15 g per day, depending on the patient’s tolerance and specific formulation. The timing and duration of GMM administration are crucial determinants of their therapeutic effectiveness. In the treatment of CDI, probiotics are often initiated alongside antibiotics and continued post-treatment to prevent dysbiosis and reduce the likelihood of recurrence (Dias et al. 2024). The duration of probiotic therapy varies from several weeks to months, depending on patient response. For *Salmonella* infections, probiotics are generally introduced at the onset of symptoms and continued for 1 to 2 weeks after symptom resolution to ensure full restoration of the gut microbiota. Prebiotics may be administered over an extended period to sustain a favorable microbial environment. In the case of FMT for CDI, a single administration is often sufficient, though some patients may require repeat treatments if symptoms persist. Despite the therapeutic potential of GMMs, their use must be carefully tailored to individual patient needs. Factors such as age, immune status, and pre-existing health conditions influence the selection of appropriate GMMs, dosages, and routes of administration. For instance, immunocompromised individuals may require reduced probiotic doses to mitigate the risk of systemic infections. Similarly, FMT necessitates rigorous donor screening to prevent the transmission of pathogenic microorganisms. Continued research is essential to refine dosing regimens and broaden the clinical applications of GMMs in the treatment of *C. difficile* and *Salmonella typhimurium* infections, ensuring both efficacy and safety in clinical practice. The limitations of existing GMM therapies for recurrent CDI play a crucial role in shaping the development of novel treatment strategies. Conventional treatments, including antibiotics, often fail to restore microbial diversity, resulting in recurrence rates of approximately 20% following initial therapy (Dieterle et al. 2019). While FMT has demonstrated effectiveness in reducing recurrence, its clinical application is hindered by challenges related to standardization and safety regulations. The recent approval of standardized microbiome restoration therapies, such as fecal microbiota, marks a significant advancement, offering improved efficacy and safety profiles in clinical evaluations (Qusty et al. 2024). Furthermore, emerging approaches—including probiotics, vaccines, and novel antibiotic formulations—seek to enhance treatment efficacy by addressing microbiome imbalances (Dieterle et al. 2019). These advancements highlight the pressing need for innovative therapeutic strategies to overcome current limitations and improve clinical outcomes in CDI management.

Specific microbial taxa and metabolic profiles serve as potential biomarkers for identifying individuals who may benefit from personalized gut microbiota modulation therapies. Studies suggest that certain bacterial groups, such as *Clostridiales* and *Roseburia inulinivorans*, are linked to enhanced responses to biological therapies in autoimmune diseases, highlighting their potential as predictive biomarkers (Qusty et al. 2024). Additionally, gut microbiota–derived metabolites play a critical role in modulating host metabolism and immune function, offering valuable insights for the development of personalized dietary interventions based on individual microbiota compositions (Abeltino et al. 2024). Moreover, integrating host genetic factors with microbiota profiling is particularly relevant in colorectal cancer management, where distinct microbial signatures may serve as predictors of treatment efficacy and adverse effects.

A clinical trial investigated the efficacy of donor feces infusion versus vancomycin in treating recurrent *Clostridium difficile* infection. The infusion group achieved an 81% resolution rate, significantly higher than vancomycin alone (31%) or vancomycin with bowel lavage (23%) (*P* < 0.001). Post-infusion, patients exhibited increased fecal bacterial diversity resembling that of healthy donors. These findings suggest that FMT is a more effective treatment for recurrent *C. difficile* infection than conventional antibiotic therapy (van Nood et al. 2013). A systematic review and meta-analysis assessed the efficacy of prebiotics, probiotics, and synbiotics in treating IBS and chronic idiopathic constipation (CIC). Analyzing 43 RCTs, probiotics significantly improved IBS symptoms (RR = 0.79, 95% CI 0.70–0.89) and increased stool frequency in CIC (mean increase = 1.49 stools/week, 95% CI 1.02–1.96). Data on prebiotics and synbiotics were limited, requiring further research. The findings suggest that probiotics are effective for IBS, but more studies are needed to clarify the role of prebiotics and synbiotics in IBS and CIC (Ford et al. 2014).

## Repurposing GMMs for neurodegenerative disorders therapy

Neurodegenerative disorders affect millions of people worldwide and are characterized by the slow progression of neuronal atrophy, leading to significant cognitive and motor impairment (Ghazy et al. 2024). These diseases predominantly appear in older adults, including conditions like AD and PD (Selmy et al. 2023). While current treatments aim to slow disease progression and improve patients’ quality of life, no curative therapies are available (Ellakwa et al. 2021). MGBA is an emerging domain of interest in AD and PD as it links the GM and CNS (Cani and Everard 2016). For instance, in AD, gut dysbiosis is thought to contribute to chronic inflammation, leading to increased amyloid-β deposition and tau hyperphosphorylation in the brain (Shaaban et al. 2022). Likewise, in PD, gut dysbiosis is linked to α-synuclein aggregation, a disease’s hallmark, which may originate in the gut and translocate to the brain via the vagus nerve (Wadan et al. 2024). Hence, the link between GM and neurodegeneration proves that GMMs could be repurposed to influence these pathological mechanisms.

A randomized, double-blind trial assessed the effects of probiotics and selenium co-supplementation on cognition and metabolism in AD patients. Seventy-nine patients received probiotics + selenium, selenium alone, or placebo for 12 weeks. The combination significantly improved cognitive function, insulin sensitivity, antioxidant levels, and lipid profiles compared to the other groups. Findings suggest probiotic and selenium co-supplementation may benefit AD, warranting further research (Tamtaji et al. 2019). Moreover, there is growing evidence that certain probiotics, such as *Lactobacillus plantarum* and *Bifidobacterium breve*, can be repurposed to treat AD and PD (Charitos et al. 2024). These probiotics may exert neuroprotective effects by reducing neuroinflammation, improving gut barrier function, and modulating brain-derived neurotrophic factor (BDNF) expression (Table 4) (Akbari et al. 2016). In mouse models of AD, administration of probiotics reduced amyloid plaque formation and improved cognitive function (Tolba et al. 2023). Similarly, in PD models, probiotics reduced α-synuclein aggregation and protected dopaminergic neurons (Ellakwa et al. 2024 d). Prebiotics like inulin and FOS can also be repurposed for neuroprotection in AD and PD by promoting the growth of beneficial GM that produces SCFAs (Akhgarjand et al. 2024). SCFAs, such as butyrate, are crucial in maintaining gut barrier integrity and reducing systemic inflammation, which are key contributors to neurodegenerative disease progression (Patrignani et al. 2024). By modulating the GM and increasing SCFA production, prebiotics offer promising adjunctive therapy for AD and PD (Baktash et al. 2018).

**Table 4:** Pharmacological modulators of gut microbiota. This table summarizes key pharmacological modulators influencing gut microbiota, including antibiotics, probiotics, prebiotics, synbiotics, antifungal treatments, and antivirals. For each modulator, details are provided on their mechanism of action, targeted microbial pathways, disease indications, clinical evidence, potential side effects, and FDA approval status. This table overviews existing treatments and their relevance in modulating the gut microbiome for therapeutic purposes

Modulator name	Mode of action	Targeted microbial pathway	Disease indication/use	Clinical evidence	FDA approval
Metronidazole	Antibiotic, disrupts DNA synthesis in anaerobic bacteria	Anaerobic bacteria, particularly in gut microbiota	Anaerobic infections, *Clostridium difficile* infections	Positive: effective against anaerobic infections, reduces gut dysbiosis	Approved for anaerobic infections and *Clostridium difficile*
Vancomycin	Inhibits cell wall synthesis in Gram-positive bacteria	Gram-positive bacteria, Clostridia species	*Clostridium difficile*, other Gram-positive infections	Positive: effective in *Clostridium difficile* treatment, restores gut balance	Approved for *Clostridium difficile* and other Gram-positive infections
Lactobacillus	Enhances gut barrier function, inhibits pathogenic bacteria	Lactobacilli, Bifidobacteria, and other beneficial gut bacteria	IBS, IBD	Positive: beneficial in improving gut health and reducing IBS symptoms	Generally regarded as safe (GRAS) for use in food supplements
Bifidobacterium	Restores gut microbiota diversity, enhances mucosal immunity	Gut microbiota diversity, potentially pathogenic bacteria	IBS, IBD, lactose intolerance, general gut health	Positive: restores gut microbiota balance, potential benefits in gut diseases	GRAS for use in food supplements
Inulin	Fermented by gut bacteria to produce SCFAs	Gut microbiota composition (increase beneficial bacteria)	Constipation, obesity, gut inflammation, diabetes	Positive: improves gut motility, beneficial in metabolic health	GRAS for use in food supplements
Fructo-oligo-saccharides	Fermented by gut bacteria to produce SCFAs, it modulates microbiota	Gut microbiota composition (increase beneficial bacteria)	Gut dysbiosis, obesity, metabolic syndrome	Positive: improves gut microbiota composition and related health outcomes	GRAS for use in food supplements
Synbiotics	Combination of probiotics and prebiotics enhances both microbial growth and immunity	Gut microbiota diversity promotes both probiotics and prebiotics	IBS, IBD, inflammatory conditions, gut dysbiosis	Positive: improves gut health, anti-inflammatory effects	GRAS for use in food supplements
Fluconazole	Inhibits fungal cell membrane synthesis	Fungal infections in the gut	Fungal infections, candidiasis	Negative: limited effectiveness in certain gut infections, potential resistance	Approved for fungal infections, candidiasis
Interferons	Modulates immune responses, potentially influences gut microbiota	Immune modulation, potential impact on gut-associated immune responses	Hepatitis, chronic viral infections, immune modulation	Positive: enhances immune responses, potential for chronic viral infection management	Approved for viral infections, including hepatitis

A randomized, double-blind clinical trial investigated the effects of probiotic supplementation on cognitive function, metabolic health, inflammation, and oxidative stress in AD patients. One study with 60 AD patients (ages 65–85) found that a 12-week multi-strain probiotic regimen (*L. acidophilus*, *L. casei*, *B. bifidum*, *B. longum*) significantly improved MMSE scores, reduced fasting glucose, LDL, and HOMA-IR, and lowered CRP, TNF-α, and IL- 6 while increasing TAC (Barbosa et al. 2017). Another trial with 40 AD patients (ages 60–85) similarly showed that probiotics reduced inflammation and oxidative stress, though cognitive improvements were not statistically significant. These findings suggest that probiotics may help in AD by modulating the gut-brain axis, reducing inflammation, and enhancing antioxidant defenses, highlighting their potential as a supportive therapy. However, further research with larger sample sizes and longer follow-up is needed to confirm long-term cognitive benefits (Andersen et al. 2016).

## Ethical considerations related to FMT in children with neurodevelopmental disorders

The use of FMT in children with neurodevelopmental disorders raises several ethical considerations that necessitate careful examination. First, obtaining informed consent is particularly challenging in this population, as parents or guardians must make decisions on behalf of children who may not fully understand the procedure due to cognitive or communicative impairments (Koh et al. 2022). This raises questions about the adequacy of proxy consent and whether parents can genuinely weigh the potential risks and benefits, especially when the evidence supporting FMT for neurodevelopmental disorders is still emerging and largely experimental (Hayashi et al. 2021). Additionally, the long-term safety of FMT in pediatric populations remains uncertain, with concerns about potential unintended consequences such as the transfer of pathogens, antibiotic resistance genes, or the disruption of the developing microbiome, which could have lasting effects on physical and neurological health (Browne et al. 2020).

Furthermore, the complex interplay between the gut microbiome and neurodevelopment is not yet fully understood, and altering the microbiome in children with neurodevelopmental disorders could lead to unpredictable outcomes, including changes in behavior, cognition, or susceptibility to other diseases (Gerber et al. 2018). Equity issues also arise, as access to FMT is often limited to high-income settings with specialized medical infrastructure, potentially exacerbating health disparities and leaving children in low- and middle-income countries without access to this potentially beneficial therapy (Gupta et al. 2021). Ethical frameworks must, therefore, emphasize rigorous safety monitoring, transparent communication with families, and equitable access to ensure that FMT is used responsibly and that its benefits are available to all patients, regardless of socioeconomic status or geographic location (Kang et al. 2017; Kassam et al. 2017). These frameworks should also address the need for ongoing research to understand better the risks and benefits of FMT in pediatric populations, particularly those with neurodevelopmental disorders, to ensure that this innovative therapy is applied in a manner that prioritizes patient well-being and minimizes harm.

## Repurposing GMMs for neuropsychiatric disorders treatment (Fig. 3)

**Fig. 3:**
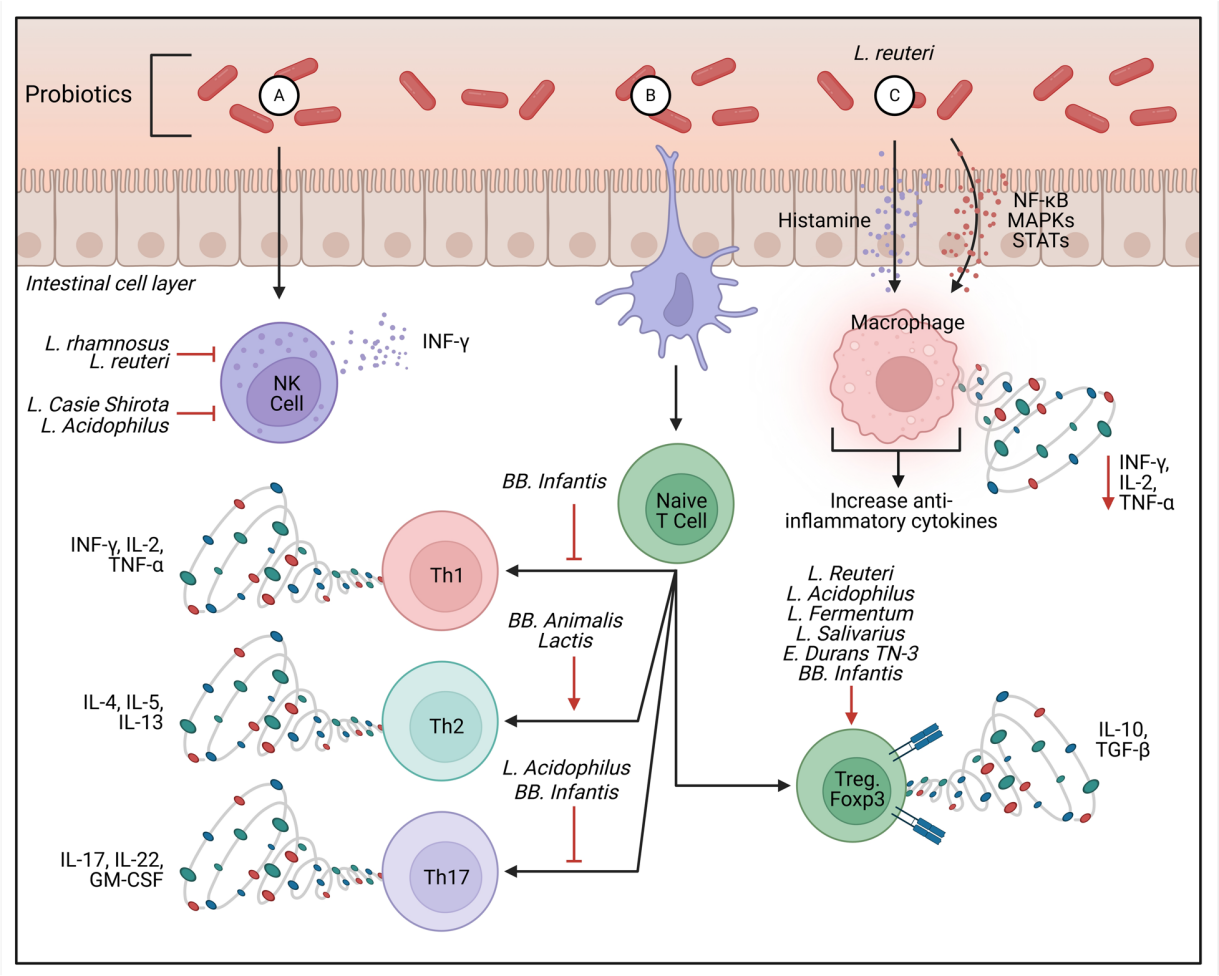
Key immunomodulatory mechanisms of probiotics in the gut that affect neuroinflammation and neuroplasticity. A Natural killer (NK) cells bridge innate and adaptive immunity, interacting with intestinal epithelial cells, dendritic cells, and T cells. Probiotics can regulate NK cell activity partly through IFN-γ secretion. B T cells play a central role in inflammation, as they can differentiate into subsets that enhance or suppress immune responses. Interactions with other immune cells, such as dendritic cells influence their differentiation. Probiotics can modulate these interactions via membrane receptors, particularly pattern recognition receptors (PRRs) like TLR- 2 and TLR- 6, which are expressed on macrophages and dendritic cells. This modulation may reduce Th17 polarization while promoting the Treg subpopulation, leading to increased IL- 10 production and decreased TNF-α levels, thereby mitigating inflammation—a key mechanism by which probiotics help manage inflammatory intestinal diseases. Additionally, probiotics appear to shift Th2-driven allergic responses toward a Th1 profile, characterized by increased IFN-γ secretion and reduced IL- 4, IL- 13, and IgE production, ultimately improving allergic conditions. Probiotics can also stimulate B cells in the lamina propria, enhancing the production of IgA, an essential immunoglobulin involved in mucosal defense. Secreted IgA binds to the mucus layer covering gut epithelial cells, providing frontline protection against gastrointestinal infections. C Probiotics also influence intracellular signaling in immune cells, such as macrophages, by modulating kinase pathways (e.g., the MAP kinase cascade), which subsequently regulate transcription factors like STAT, NF-κB, Jun- 1, and Fos. Additionally, probiotic metabolism of histamine can affect antigen-presenting cells via H2 receptors, reducing pro-inflammatory cytokines such as TNF-α, IL- 12, and monocyte chemotactic protein- 1 (Soliman and Abdellatif 2023)

Gut dysbiosis, or imbalance in GM, has been linked to neuropsychiatric disorders such as attention-deficit hyperactivity disorder (ADHD) and autism spectrum disorder (ASD) (Kunde et al. 2013). ADHD is often associated with gastrointestinal issues like constipation and abdominal cramps, as well as conditions like food allergies and eczema, which are influenced by GM and immune factors (Millan et al. 2018). Likewise, in ASD, increased intestinal permeability alters gut homeostasis, leading to changes in SCFA levels (Sharon et al. 2019). Traditionally, FMT has been used to treat gastrointestinal and metabolic conditions (Wang et al. 2023). Considering the MGBA, there is growing interest in repurposing these agents for neurodevelopmental conditions like ASD and ADHD, where microbial imbalances and gut dysfunctions are often observed (Kim et al. 2022). For example, microbiota transfer therapy and specific probiotics have shown the potential to modulate social behaviors in ASD. However, individual variability in microbiota composition suggests that personalized approaches may be necessary to optimize efficacy (Principi et al. 2018). FMT has been linked to increases in *Akkermansia muciniphila* and reductions in TNF-α levels, which are associated with reduced neuroinflammation and potentially beneficial in the context of ASD (Akram et al. 2024). Similarly, specific probiotic strains like *Lactobacillus reuteri* have shown the potential to enhance social behaviors in animal models of ASD, possibly through mechanisms involving oxytocin modulation (Nobre et al. 2022). Additionally, GMMs may influence neurotransmitter levels and neurotrophic factors relevant to ASD and ADHD (Lamptey et al. 2022). Elevated BDNF has been associated with improvements in neuroplasticity, cognition, and behavior, while imbalances in neurotransmitters such as glutamate and serotonin are frequently observed in ASD (Ahmed et al. 2020). Probiotics, such as *Lactobacillus* and *Bifidobacterium* species, can impact the secretion of these neuroactive compounds, thereby offering therapeutic options for alleviating ASD and ADHD symptoms (Palanisamy et al. 2023). A clinical trial on ASD found that modifying the gut microbiota may improve GI and behavioral symptoms. An open-label trial of MTT combining antibiotics, bowel cleanse, stomach-acid suppressant, and fecal microbiota transplant showed lasting GI improvements, continued autism-related symptom relief, and increased gut microbiota diversity. A 2-year follow-up confirmed these benefits, supporting MTT’s long-term safety and efficacy and the need for placebo-controlled trials (Palanisamy et al. 2023).

Current research on the application of GMMs for neuropsychiatric disorders faces several limitations. Firstly, heterogeneity in study designs—including differences in probiotic strains, dosages, and treatment durations—hinders the ability to draw definitive conclusions regarding their efficacy (Megur et al. 2020). Secondly, most existing studies are short-term, leaving the long-term safety and effectiveness of GMM interventions uncertain (Cheng et al. 2019a). Thirdly, individual variability in baseline gut microbiota composition contributes to inconsistent responses to GMMs, complicating the establishment of universal treatment protocols (Sochocka et al. 2019). These challenges underscore the necessity for standardized, long-term research to comprehensively evaluate the therapeutic potential of GMMs in neuropsychiatric disorder management.

## Repurposing GMMs for cancer treatment (Fig. 4)

**Fig. 4:**
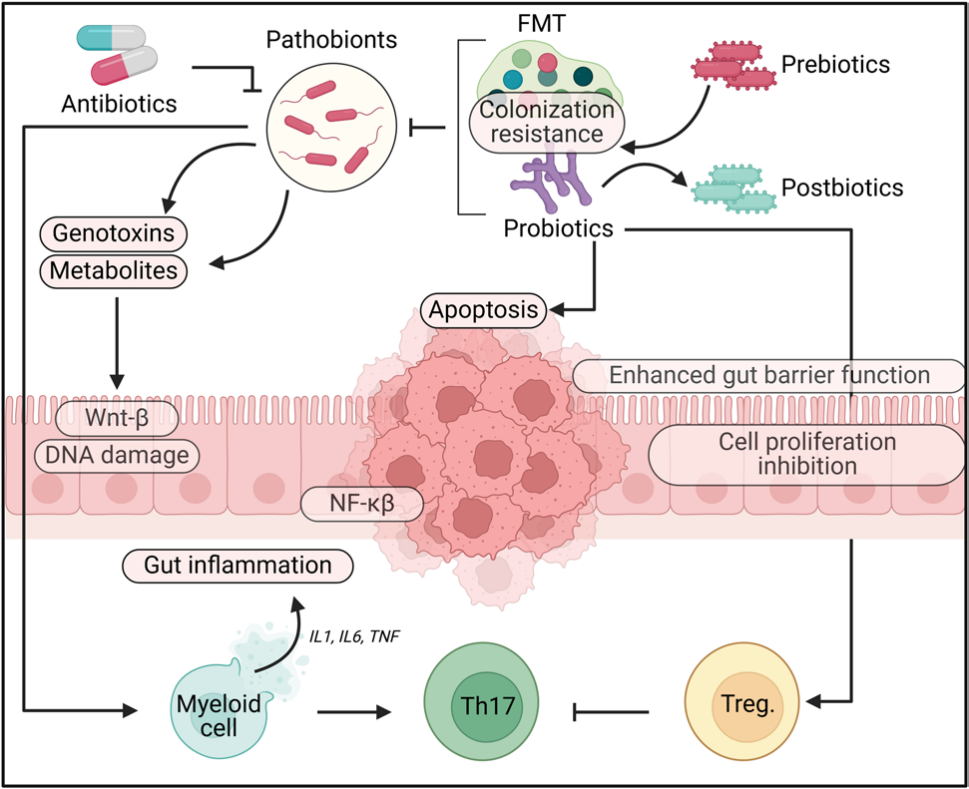
The gut microbiota can impact carcinogenesis through various mechanisms, including microbial-derived factors such as metabolites or genotoxins. Disrupted host-microbe interactions activate pro-carcinogenic inflammatory pathways, leading to cancer progression. While antibiotics effectively eliminate pathobionts, their broad antimicrobial effects can disturb gut balance by targeting beneficial bacteria, limiting their use in CRC treatment. Prebiotics support the growth of probiotics. Probiotics influence cancer prevention through multiple mechanisms. They can prevent pathogenic bacteria colonization, enhance barrier function by increasing mucin production and tight junction protein expression, promote immune responses that boost anti-inflammatory actions of Treg cells, modulate pro-inflammatory cytokine release, and induce apoptosis in cancer cells. Postbiotics selectively target tumor cells, controlling their proliferation by inhibiting NFATc3 activation. Lastly, fecal microbiota transplantation (FMT) may help manage CRC by restoring microbiome balance and promoting immune responses; however, potential risks of FMT include the introduction of new pathobionts and the spread of disease-related genes

The gastrointestinal microbiota significantly influences cancer treatment outcomes, including chemotherapy, immunotherapy, and radiation therapy (Sochocka et al. 2019). GMMs used in gastrointestinal disorders can be repurposed as drug adjuvants to improve immunotherapy response (Fitzgerald et al. 2019). For example, a study demonstrated that a consortium of 11 microbial strains enhanced the effectiveness of immune checkpoint inhibitors (ICIs) in mice by activating CD8 + T cells through CD103 + dendritic cells (Ojha et al. 2023). Probiotics like *Bifidobacterium lactis* and *Lactobacillus acidophilus* enhance butyrate-producing *Faecalibacterium* and improve immunotherapy outcomes in colorectal cancer. Prebiotics such as inulin and galacto-oligosaccharides promote immune-stimulating bacteria, and high-fiber diets further increase *Bifidobacterium* levels, benefiting metastatic lung cancer patients undergoing ICIs (Duan et al. 2015).

**Fig. 5:**
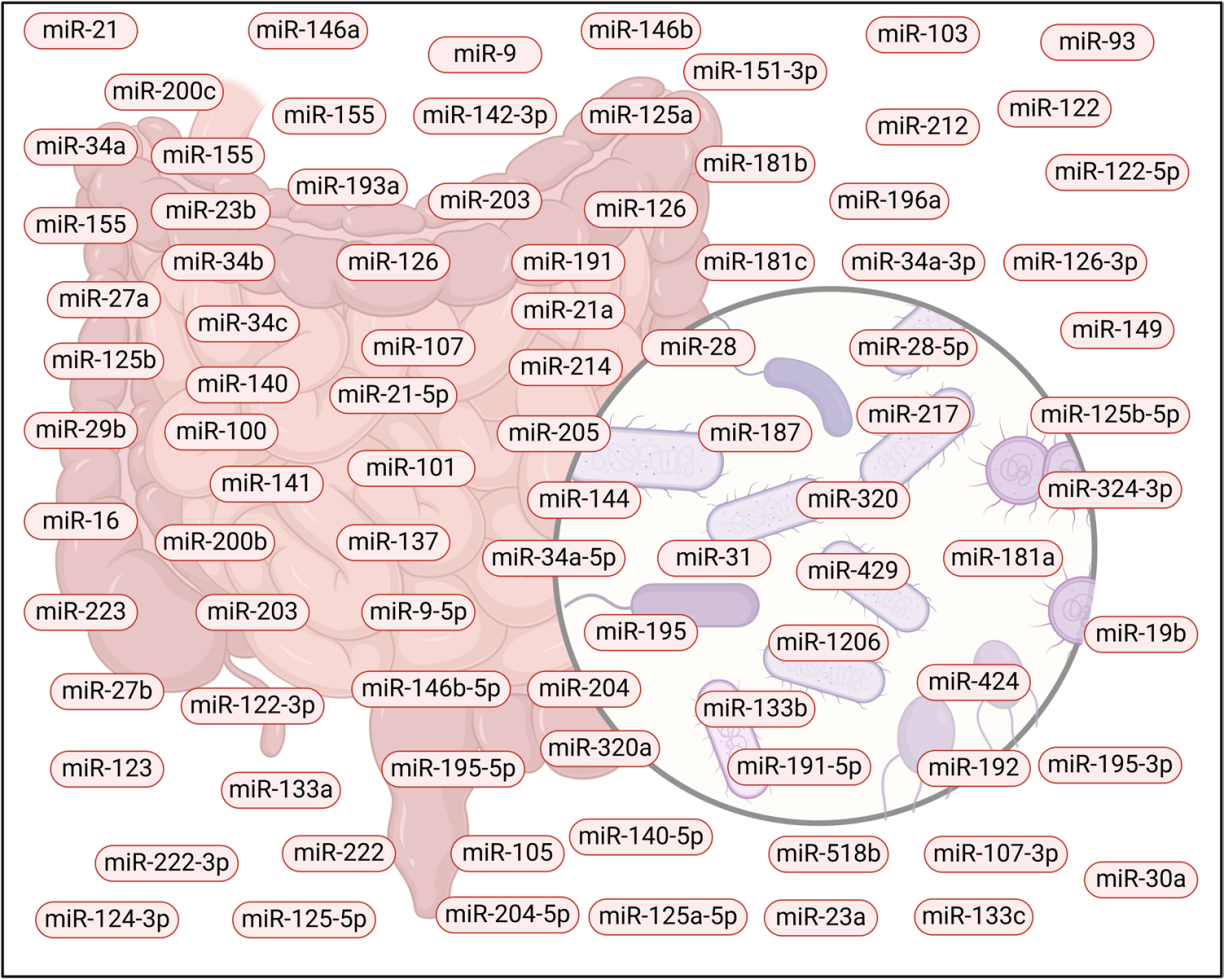
MicroRNAs are involved in various cancers like colorectal, gastric, liver, breast, and pancreatic cancers and reflect their involvement in the modulation of the gut microbiota

Recent research has shown that microRNAs (miRNAs) play a crucial role in modulating the gut microbiome by influencing various biological processes, including inflammation, immune responses, gut barrier integrity, and metabolism (Fig. 5). miRNAs like miR- 21 and miR- 155 regulate inflammatory pathways that can shift microbial composition toward a pro-inflammatory environment, while miR- 146a and miR- 223 influence gut immune responses and microbial homeostasis (Abdelhamid et al. 2022). miRNAs such as miR- 200c and miR- 29b affect gut barrier integrity by regulating epithelial functions and tight junction proteins, impacting how microbes interact with the gut epithelium. Additionally, miRNAs like miR- 34a and miR- 122 regulate metabolic pathways, influencing gut microbial diversity and functionality. In disease contexts, miRNAs such as miR- 200b and miR- 181b modulate gut microbiota in conditions like colorectal cancer and IBD by influencing inflammation and immune responses. Stress-related changes in the gut microbiome are also regulated by miRNAs like miR- 9 and miR- 26b, which affect the brain-gut axis. Overall, miRNAs are integral to maintaining gut microbiome balance and contribute to disease progression by modulating microbial composition and host responses (Kaur et al. 2020).

## GMMs in cancer prevention: mechanisms and potential

Genetically modified microbes hold significant potential in cancer prevention by leveraging their ability to modulate the gut microbiota and interact with host physiology. One key mechanism involves reducing chronic inflammation, a well-known driver of carcinogenesis. By promoting the growth of anti-inflammatory bacteria such as *F. prausnitzii* and *Bifidobacterium* species, GMMs can enhance the production of SCFAs, particularly butyrate, which suppresses NF-κB signaling and mitigates inflammation-driven cancers like colorectal cancer (Gazerani 2019). Beyond inflammation control, GMMs contribute to cancer prevention by neutralizing carcinogens. *Lactobacillus* and *Bifidobacterium* species have demonstrated the ability to degrade carcinogenic compounds like heterocyclic amines and polycyclic aromatic hydrocarbons commonly found in processed foods and pollutants (Kang and Zivkovic 2021). Similarly, *E. limosum* metabolizes dietary nitrosamines, potent carcinogens linked to gastric cancer (Woodworth et al. 2017). Engineering GMMs to enhance these detoxification pathways could further reduce carcinogen exposure and lower cancer risk.

Another crucial aspect of cancer prevention is maintaining gut barrier integrity. A weakened gut barrier allows harmful bacterial byproducts, such as LPS, to enter systemic circulation, triggering inflammation and immune dysregulation. GMMs strengthen the gut barrier by promoting mucus production, tight junction proteins, and beneficial bacterial growth. *A. muciniphila* enhances gut integrity and reduces systemic inflammation, potentially lowering cancer risk (Kang et al. 2019), while *B. fragilis* produces polysaccharide A, further reinforcing the gut barrier and modulating inflammation (Cheng et al. 2019a). GMMs also enhance immune surveillance, improving the body’s ability to detect and eliminate precancerous cells. By shaping the gut microbiota, GMMs stimulate Tregs and dendritic cells, which maintain immune homeostasis and prevent abnormal cell growth (Sarkar et al. 2016). Additionally, Bifidobacterium species enhance NK cell activity, which is crucial for identifying and destroying cancer cells (Cheng et al. 2019a). These mechanisms, reducing chronic inflammation, neutralizing carcinogens, strengthening the gut barrier, and modulating immune surveillance, highlight the preventive potential of GMMs, especially in high-risk populations. While further research is needed to refine their design, delivery, and long-term safety, GMMs could become a key tool in preventive healthcare, offering an innovative approach to reducing the global cancer burden.

## The potential for GMMs to reduce the side effects of cancer treatments

GMMs offer a promising approach to mitigating several adverse effects associated with cancer treatments, thereby enhancing patient comfort and treatment adherence. Probiotics such as *Lactobacillus rhamnosus* have demonstrated efficacy in reducing the severity of chemotherapy-induced mucositis, a debilitating inflammation affecting the oral and gastrointestinal mucosa (Alexander et al. 2017). This protective effect is attributed to the probiotic’s ability to reinforce the mucosal barrier, attenuate inflammation, and facilitate tissue repair, ultimately alleviating symptoms and enhancing patient quality of life. Similarly, in the context of radiation therapy, where disruptions to gut microbiota frequently result in complications such as radiation-induced diarrhea, interventions like FMT and specific probiotics help restore microbial balance, thereby reducing both the incidence and severity of gastrointestinal toxicity (Gopalakrishnan et al. 2018). By replenishing beneficial bacteria and restoring microbial diversity, these strategies help maintain gut integrity and function, which are critical for minimizing radiation-related side effects.

Beyond gastrointestinal complications, GMMs have the potential to modulate immune responses, thereby mitigating immune-related adverse events (irAEs) associated with immunotherapy, such as colitis and hepatitis (Mager et al. 2020). ICIs can provoke excessive immune activation, leading to inflammatory damage in various organs. Through their ability to regulate immune cell activity and produce anti-inflammatory metabolites such as SCFAs, GMMs can help suppress these dysregulated immune responses. Butyrate-producing bacteria, for instance, have been shown to reduce gut inflammation, thereby alleviating colitis, while other microbial metabolites play a role in modulating systemic immune responses to prevent hepatitis and other irAEs. In addition to addressing these specific adverse effects, GMMs hold broader potential for improving cancer treatment tolerability. They may help alleviate chemotherapy-induced nausea and vomiting by stabilizing the gut-brain axis and reducing neuroinflammation (Matson et al. 2018). Moreover, GMMs can enhance the bioavailability of specific chemotherapeutic agents by modulating gut microbial enzymes, potentially allowing for lower dosages and reducing the risk of associated toxicities (Sivaprakasam et al. 2016). By effectively mitigating these side effects, GMM-based interventions not only enhance the quality of life for cancer patients undergoing treatment but also promote treatment adherence, thereby optimizing therapeutic outcomes.

## Repurposing GMMs as drug carriers

Microbiota-responsive drug delivery systems are specifically engineered to release therapeutic compounds in reaction to particular alterations in the GM (Zitvogel et al. 2018a). These systems may be designed to identify and react to microbial byproducts, pH fluctuations, or bacterial populations (Zitvogel et al. 2018b). Currently used to maintain a balanced GM, GMMs such as probiotics can be engineered to carry and release therapeutic compounds at specific sites within the gut (Routy et al. 2018). This approach enables targeted delivery of medications such as anti-inflammatory agents or cancer therapies, repurposing probiotics from simple gut health aids to precision delivery vehicles (Viaud et al. 2013). GMMs also minimize off-target effects by releasing active agents specifically at desired sites, such as the gut or microbiota-influenced organs, which reduces unintended impacts on non-target tissues (Fitzgerald et al. 2019). Additionally, as naturally biocompatible agents, they lower the risk of immune response and toxicity, providing a safer alternative to synthetic delivery systems (Iida et al. 2013).

Numerous studies have investigated the potential of GMMs as drug carriers, demonstrating their ability to transform targeted drug delivery across various diseases. Engineered *E. coli Nissle* 1917, for instance, has been utilized to deliver anti-inflammatory cytokines, such as IL- 10, in preclinical models of IBD. By leveraging the probiotic’s capacity to colonize the gut and produce IL- 10 locally, this approach effectively reduces inflammation while avoiding the systemic side effects associated with conventional cytokine therapies (Aggarwal et al. 2022). Similarly, advancements in cancer research have explored the use of engineered Lactobacillus strains to deliver chemotherapeutic agents directly to tumors in mice. A 2022 study demonstrated that these strains, designed to release drugs in response to the tumor microenvironment’s unique conditions—such as low oxygen levels—significantly reduced systemic toxicity while enhancing therapeutic efficacy (Duan et al. 2015).

The application of GMMs as drug carriers has further expanded with recent trials exploring novel therapeutic strategies. In autoimmune disease research, engineered *Bifidobacterium longum* has been used to deliver anti-tumor necrosis factor (TNF) antibodies in preclinical models of rheumatoid arthritis, leading to significant reductions in joint inflammation and damage, highlighting the precision of GMM-based treatments (Koppel et al. 2018). In cancer therapy, engineered *Salmonella typhimurium* has been designed to produce cytotoxic agents in response to hypoxic conditions within tumors, effectively reducing tumor growth in mouse models while minimizing damage to healthy tissues (Louis et al. 2014). Furthermore, innovative approaches in metabolic disorders have been explored, such as a 2021 study investigating *Saccharomyces boulardii* engineered to produce glucagon-like peptide- 1 (GLP- 1) for the treatment of type 2 diabetes. This intervention significantly improved glucose tolerance and insulin sensitivity in preclinical models, presenting a promising new strategy for managing metabolic diseases (Plovier et al. 2017).

Beyond genetic modifications, surface engineering techniques have been employed to improve targeted drug delivery by enabling probiotics to express adhesion proteins, which enhance their ability to bind to specific tissues. *Lactobacillus reuteri*, for example, has been engineered to express intestinal epithelial cell adhesion proteins, allowing it to localize more effectively to inflamed gut tissues in IBD models. This targeted approach increases the local concentration of therapeutic agents while reducing off-target effects, ultimately enhancing treatment efficacy (Round and Mazmanian 2010). Additionally, synthetic biology has revolutionized GMM-based drug delivery by enabling the design of genetic circuits that trigger the release of therapeutic compounds in response to environmental cues such as pH changes, inflammation, or microbial metabolites. A recent 2024 study demonstrated how engineered *Bifidobacterium longum* could release anti-cancer drugs in response to the acidic tumor microenvironment, ensuring that drug delivery occurs exclusively at the tumor site (Schwabe and Jobin 2013). This pH-sensitive genetic circuit minimizes systemic toxicity while maximizing therapeutic impact, highlighting the potential of GMMs in precision medicine.

GMMs have demonstrated significant potential in treating neurological disorders, ocular diseases, and viral infections by enabling targeted drug delivery. For instance, engineered *L. rhamnosus-*producing GABA exhibited anxiolytic effects in preclinical models, offering a promising approach for anxiety management (Viaud et al. 2013). Similarly, GMMs engineered to produce 5-HTP, a precursor to serotonin, have shown potential for treating mood disorders by modulating the gut-brain axis (Bach Knudsen et al. 2018). Beyond neurological applications, engineered *B. breve* was utilized for systemic VEGF inhibitor delivery, reducing abnormal blood vessel growth in AMD models by responding to oxidative stress, a hallmark of the disease (Peterson 2020). This highlights the potential of GMM-based oral therapies for ocular disorders. Furthermore, the ability of GMMs to combat viral infections was demonstrated using engineered *Lactobacillus* strains capable of releasing antiviral peptides in response to viral particles, effectively reducing rotavirus and norovirus loads in preclinical models (Cenit et al. 2017). These interconnected studies underscore the broad therapeutic scope of GMMs, spanning neurological, ocular, and infectious diseases through precision-targeted interventions.

## Challenges and limitations for using GMMs for drug delivery

While GMMs hold immense promise as targeted drug delivery systems, several challenges must be addressed to harness their therapeutic potential fully. One of the primary obstacles is ensuring their survival and functionality in the harsh environment, where fluctuating pH levels, bile salts, and competition from native microbiota can significantly reduce their viability. For instance, the acidic conditions of the stomach often compromise the survival of orally administered GMMs before they reach the intestines, necessitating protective strategies such as encapsulation techniques and genetic modifications to enhance resistance (Boonchooduang et al. 2020; Khanna et al. 2022). Beyond survival, precise control over the timing and location of drug release is essential for maximizing therapeutic efficacy while minimizing off-target effects. Synthetic biology approaches have enabled the engineering of genetic circuits that respond to disease-specific biomarkers, such as hypoxia in tumors or inflammation in the gut, to regulate drug release [129]. However, variability in gut microbiota composition and host environmental conditions complicates this approach, highlighting the need for personalized therapies and robust genetic designs that function reliably in vivo (Mahmoudi and Hossainpour 2023).

The host immune response further challenges the clinical translation of GMMs, as engineered microbes may be recognized as foreign and rapidly eliminated. Immune cells such as macrophages and dendritic cells can reduce the colonization and therapeutic effectiveness of GMMs, prompting researchers to explore immune-evasive strategies, including surface protein modifications and immune-conditioning techniques (Yenkoyan et al. 2024; Cickovski et al. 2023). In addition to biological and engineering hurdles, regulatory and safety concerns present significant barriers to widespread adoption. The potential for unintended gene transfer to native microbiota, off-target therapeutic effects, and long-term ecological impacts necessitate rigorous evaluation and the establishment of standardized guidelines for clinical use (Zhang et al. 2023). Finally, large-scale production of GMMs poses additional challenges, requiring optimization of manufacturing, storage, and delivery methods to ensure consistent quality, stability, and potency from production to administration (Sgritta et al. 2019).

## Future directions in GMM-based drug delivery systems

The field of GMMs for drug delivery is rapidly advancing, with emerging research poised to revolutionize disease treatment. By overcoming current limitations and leveraging cutting-edge technologies, researchers can fully harness GMMs as precision therapeutic tools. One promising approach is the development of “smart” probiotics, engineered to respond to real-time gut environment dynamic changes, enabling more precise, context-dependent drug release. Advances in synthetic biology and genetic engineering now allow the creation of GMMs with sophisticated genetic circuits that sense and react to specific biomarkers, such as pH, inflammation, or microbial metabolites (Ilchibaeva et al. 2023). For example, engineered microbes could release anti-inflammatory agents only when detecting elevated pro-inflammatory cytokines, ensuring treatment is delivered only when and where needed. Additionally, these self-regulating probiotics could shut down drug production once the target condition resolves, minimizing over-treatment risks and side effects.

Another key advancement is the development of personalized GMM therapies, optimized for an individual’s microbiota composition to enhance therapeutic efficacy. Since gut microbiota varies significantly among individuals, tailoring GMMs to specific microbial environments could improve colonization efficiency and treatment outcomes (Cheng et al. 2019b). IBD, personalized GMMs could be designed to produce anti-inflammatory cytokines or repair gut barrier function based on a patient’s immune profile. Similarly, in cancer treatment, GMMs could deliver chemotherapeutic agents or immune modulators tailored to the tumor microenvironment, reducing adverse effects and improving response rates. Integrating GMMs with nanotechnology presents another frontier in precision drug delivery, enhancing stability, targeting, and controlled drug release. Nanoparticles improve therapeutic agent stability, while GMMs offer a biocompatible delivery system, ensuring specific site targeting within the body. Engineered GMMs could be designed to produce and release drug-loaded nanoparticles in response to environmental cues, such as tumor acidity or gut inflammation (Paudel et al. 2024). This combined approach would improve treatment precision, allowing for lower drug doses and reduced systemic toxicity. Furthermore, nanotechnology could protect GMMs from harsh gut conditions, improving survival and colonization.

While most GMM research has focused on the gut, expanding their applications to other microbiota-influenced organs, such as the liver, brain, and skin, could unlock new treatment avenues. The gut-brain and gut-liver axes play a crucial role in disease pathogenesis, suggesting the potential for GMMs in neurological and metabolic disorders (Chrysostomou et al. 2023). In neurodegenerative diseases like Parkinson’s or Alzheimer’s, engineered GMMs could modulate neurotransmitter levels or produce neuroprotective compounds, offering non-invasive therapeutic options. Similarly, targeting the gut-liver axis could allow GMMs to regulate systemic inflammation and insulin sensitivity, providing innovative solutions for metabolic syndrome. As GMM-based therapies progress toward clinical application, addressing safety and regulatory challenges will be essential. Ensuring the long-term stability and persistence of engineered microbes, minimizing unintended gene transfer, and assessing the ecological impact of introducing GMMs into the human body are critical considerations (Abbas et al. 2022a). Rigorous clinical trials and standardized regulatory frameworks will be necessary to confirm the safety and efficacy of these innovative therapies, paving the way for widespread clinical adoption.

## Applications of GMMs repurposing

### Pharmacological repurposing

**Fig. 6:**
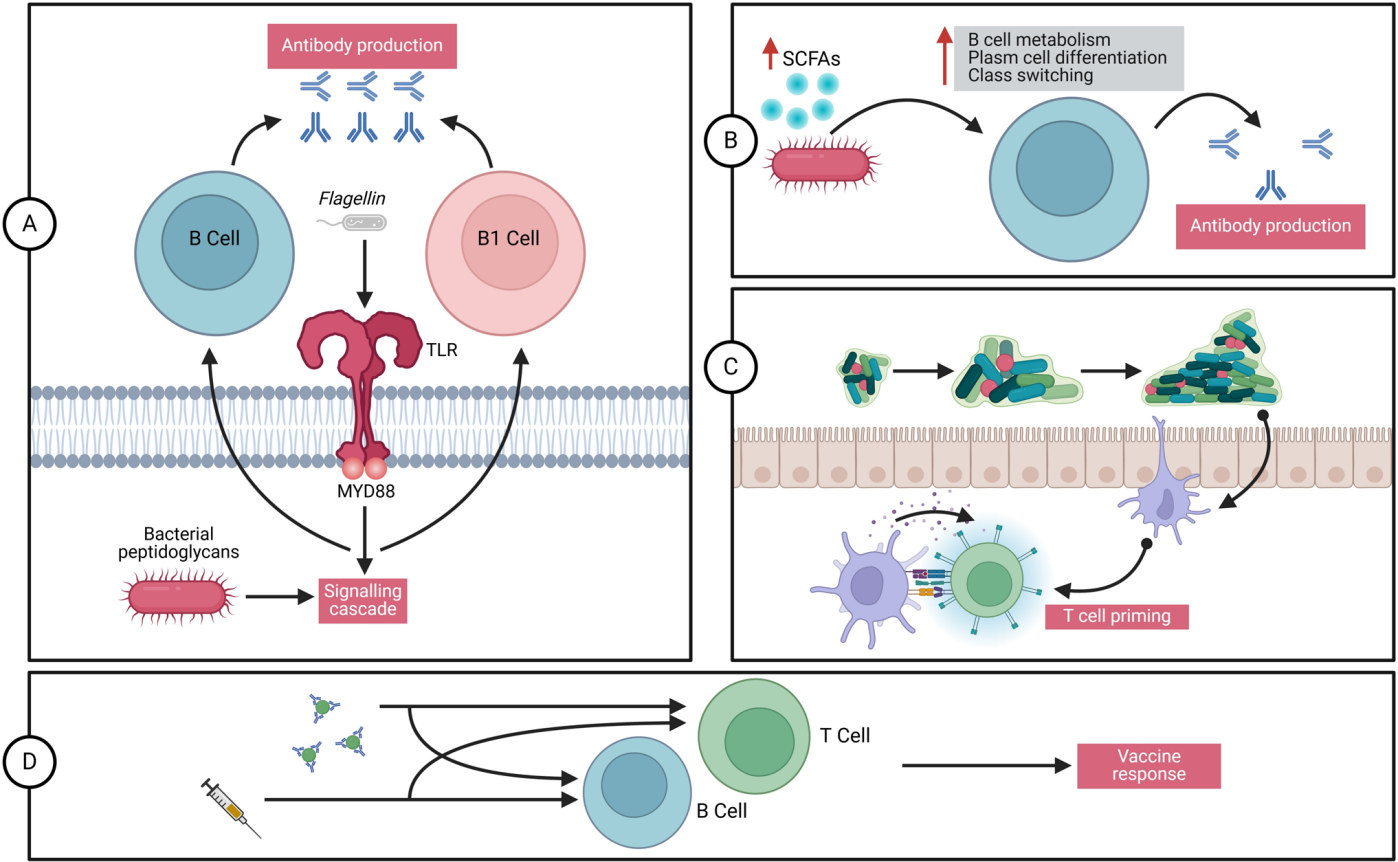
Immunomodulatory effects of the microbiota on vaccine responses. **A** Microbiota-derived immunomodulatory molecules, such as flagellin and peptidoglycan, function as natural adjuvants that can enhance vaccine responses. In animal models, these molecules are recognized by PRRs, including TLRs, which APCs express. Other microbial components, such as lipopolysaccharides, may similarly influence immune responses. PRRs found on T and B cells may also detect these molecules directly, contributing to immune modulation. **B** DCs play a key role in vaccine-induced immune responses by presenting antigens to T cells and secreting immunomodulatory cytokines. The microbiota influences the production of interferons by dendritic cells, which subsequently shape the metabolic and epigenetic states of conventional dendritic cells, enhancing their ability to prime T cells. **C** Microbiota-derived metabolites, such as SCFAs, support B cell metabolism by meeting the high energy demands required for antibody production. These metabolites also upregulate genes involved in plasma cell differentiation and class switching, potentially modifying vaccine responses. **D** Emerging evidence suggests microbiota-derived antigens may be cross-reactive with those encoded by pathogens or vaccines. Cross-reactive B cells or T cells could influence immune responses to vaccination

Pharmacological repurposing accelerates the discovery of new therapeutic applications for existing drugs, reducing development time and costs (Tanoue et al. 2019). Integrating GMMs with drug delivery systems offers cost-effective treatment options by improving manufacturing processes, increasing yield, and decreasing reliance on traditional chemical synthesis, thus promoting sustainability and accessibility in drug development (Hibberd et al. 2017). The global probiotics market is projected to exceed USD 3 billion by 2024, driven by increased consumer awareness of GMM-related health benefits (Zhang et al. 2021a). GMMs also play a critical role in modulating the immune system’s response to vaccines (Fig. 6) (Xie et al. 2022).

Specific microbial metabolites, including probiotics and prebiotics, act as natural adjuvants, enhancing immune responses and potentially leading to safer and more effective vaccines (Aggarwal et al. 2020a). This is especially relevant for populations with varying vaccine responses, such as the elderly and immunocompromised individuals (Kumar et al. 2016). Moreover, GMMs can improve the efficacy of vaccine adjuvants, which are key to many vaccines (Zhao et al. 2020a). Additionally, as mentioned earlier, GMM drug delivery systems have been developed to release therapeutics based on specific changes in GM, such as shifts in pH or microbial metabolites, and it can be utilized to ensure precise, timely vaccine delivery, minimizing side effects and improving overall efficacy (Tong et al. 2020). Evidence from current animal models confirms the influence of GMMs on immune responses to vaccination (Parvathaneni et al. 2019a). In one study, mice treated with antibiotics or raised in germ-free (GF) conditions exhibited heightened IgG and IgA responses to an orally administered mouse rotavirus, which serves as a model for oral rotavirus vaccines (Kawale et al. 2024). Another study reported that reintroducing the normal microbiota into GF mice enhanced their immune responsiveness following systemic immunization. In contrast, GF and antibiotic-treated conventional mice exhibited decreased serum antibody and T cell responses compared to age-matched conventional controls (Abbas et al. 2022a).

### Non-pharmacological repurposing

Non-pharmacological gut microbiota modulators, including dietary modulation, FMT, exercise, sleep hygiene, stress management, and gastric bypass surgery, play a significant role in maintaining or restoring a healthy gut microbiome (Table 5). These interventions help promote the growth of beneficial bacteria, reduce pathogenic microbes, and improve gut function. For example, a fiber-rich diet supports beneficial bacteria and reduces inflammation, while FMT restores microbial diversity, especially in conditions like *Clostridium difficile* infection (Ciabattini et al. 2019; Huang et al. 2023). Exercise enhances gut microbiota diversity and promotes metabolic health, while good sleep hygiene improves gut barrier function. Stress management and mindfulness help reduce gut inflammation and improve the gut-brain axis. Gastric bypass surgery induces favorable microbial shifts that improve metabolic health and aid in weight loss (Rossouw et al. 2024).

**Table 5:** Nonpharmacological modulators of gut microbiota

Intervention type	Mechanism of action	Impact on microbial composition	Health benefits/applications	Scientific evidence/clinical studies
dietary modulation	Increases fiber intake, promotes beneficial bacteria growth	Increases abundance of beneficial bacteria like Bifidobacteria and Firmicutes	Improves gut health, reduces inflammation, enhances immune function	Positive: demonstrated improvements in gut health through various diet studies
FMT	Restores microbiota balance by transferring fecal matter from healthy individuals	Restores diversity and enhances beneficial bacteria, reducing harmful pathogens	Improves gut health, aids in the treatment of gut disorders (e.g., *C. difficile* infection)	Positive: clinical success in treating *C. difficile* infections, positive effects on microbiota
Exercise and physical activity	Promotes beneficial microbiota growth through physical activity and metabolic changes	Increases diversity of gut microbiota, improves microbiome stability	Improves metabolic health, reduces inflammation, aids in gut disorders	Positive: exercise shown to improve gut health and metabolic profiles in multiple studies
Sleep hygiene	Regulates gut health by improving circadian rhythms and gut microbiota composition	May normalize microbial composition through improved metabolism and sleep cycles	Improves gut and mental health, reduces inflammation, aids in metabolic balance	Positive: evidence supports improved microbiota composition and metabolism with quality sleep
Stress management and mindfulness	Reduces gut inflammation and balances the gut-brain axis	Improves gut health by reducing inflammation and potentially enhancing microbiome balance	Improves gut health, mental health, reduces inflammation, regulates the gut-brain axis	Positive: studies indicate significant improvements in microbiota balance and mental health
Gastric bypass surgery	Affects gut microbial composition, potentially beneficial for metabolic health	Promotes microbial shifts towards a more favorable composition post-surgery	Improves metabolic health, reduces inflammation, beneficial for weight loss and obesity	Positive: evidence suggests benefits for metabolic health and gut microbiota post-surgery

## Pharmacological repurposing

### Fecal microbiota transplantation

**Fig. 7:**
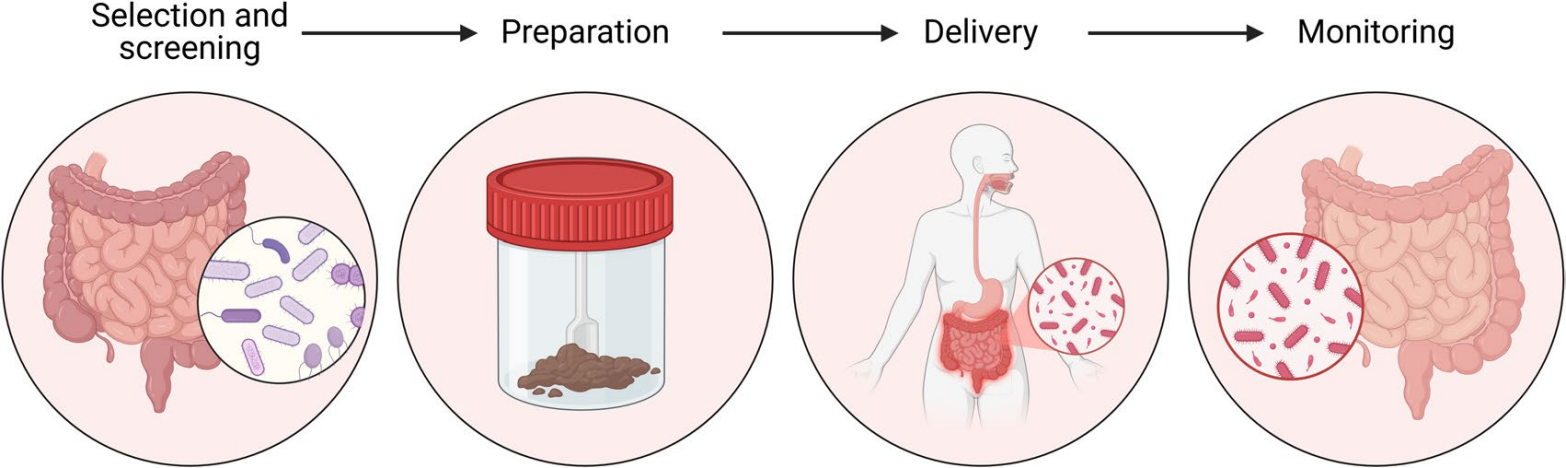
The FMT process involves (1) donor selection and screening, (2) preparation of bacterial suspension and freeze-dried capsules, (3) administration through upper gastrointestinal routes (nasogastric tube, gastroscopy) or lower gastrointestinal routes (colonoscopy, sigmoidoscopy), and (4) careful post-transplant monitoring

FMT, the microbiota transfer from a screened donor to a recipient, has emerged as a revolutionary strategy to modulate the gut microbiome and restore health (Fig. 7). FMT exerts its effects through three primary mechanisms: colonization of donor microbes, restoration of microbial diversity, and modulation of host immunity and metabolism. Upon transplantation, donor-derived bacteria colonize the recipient’s gut, competing with pathogenic microbes for resources and niche spaces. For instance, in CDI, donor *Bacteroidetes* and *Lachnospiraceae* species inhibit *C. difficile* growth by producing SCFAs like butyrate, suppressing pathogen proliferation. Additionally, FMT reintroduces microbial enzymes and metabolites that enhance metabolic pathways, such as tryptophan metabolism, which regulates gut barrier function and anti-inflammatory responses. FMT has proven most effective in treating recurrent CDI, achieving cure rates exceeding 90% in clinical trials. Its success in this context has spurred exploration in other gastrointestinal and extraintestinal conditions. In IBD, FMT shows moderate efficacy, particularly when donor-recipient microbiota compatibility is optimized. For example, studies report remission rates of 20–50% in ulcerative colitis (UC), contingent on donor microbial composition and engraftment success. Beyond gastrointestinal disorders, FMT is being investigated for its role in metabolic health. Preclinical and clinical data suggest that FMT from lean donors may improve insulin sensitivity and weight management in obese recipients, though long-term efficacy remains debated. In aging populations, FMT from young donors has been proposed to rejuvenate the gut microbiome, potentially delaying age-related diseases by restoring microbial diversity and SCFA production (Zimmermann 2023).

Despite its promise, FMT faces significant hurdles. Donor selection is critical, as microbiota composition varies widely among individuals. “Super-donors” with highly diverse and resilient microbiomes are sought, but standardized criteria for donor screening remain elusive. Safety concerns also persist; FMT carries risks of pathogen transmission, as exemplified by a 2019 case of *Escherichia coli* infection linked to untested stool. Regulatory frameworks must ensure rigorous donor screening and stool banking protocols. Engraftment variability further complicates outcomes. The recipient’s native microbiota often resists colonization by donor microbes, a phenomenon termed colonization antagonism. Factors such as diet, antibiotic use, and recipient immune status influence engraftment success, necessitating personalized approaches. Finally, ethical and cultural barriers, including those associated with stool donation, may hinder FMT acceptance (Lynn et al. 2022).

### Bacteriophage

Bacteriophages—viruses that infect bacteria—are Earth’s most abundant biological entities, yet their roles in the gut microbiome remain enigmatic. While traditionally studied for combating harmful bacteria, phages also target commensal bacteria, influencing microbiome structure and health. Research links distinct phage populations to IBD and other gut disorders. For example, a study reanalyzing samples from the TEDDY cohort found dynamic changes in phage-bacteria communities during early life, suggesting an “arms race” between bacteria and phages as they evolve to evade each other. This interplay may impact immune system development and disease susceptibility (Uchiyama et al. 2014).

**Fig. 8:**
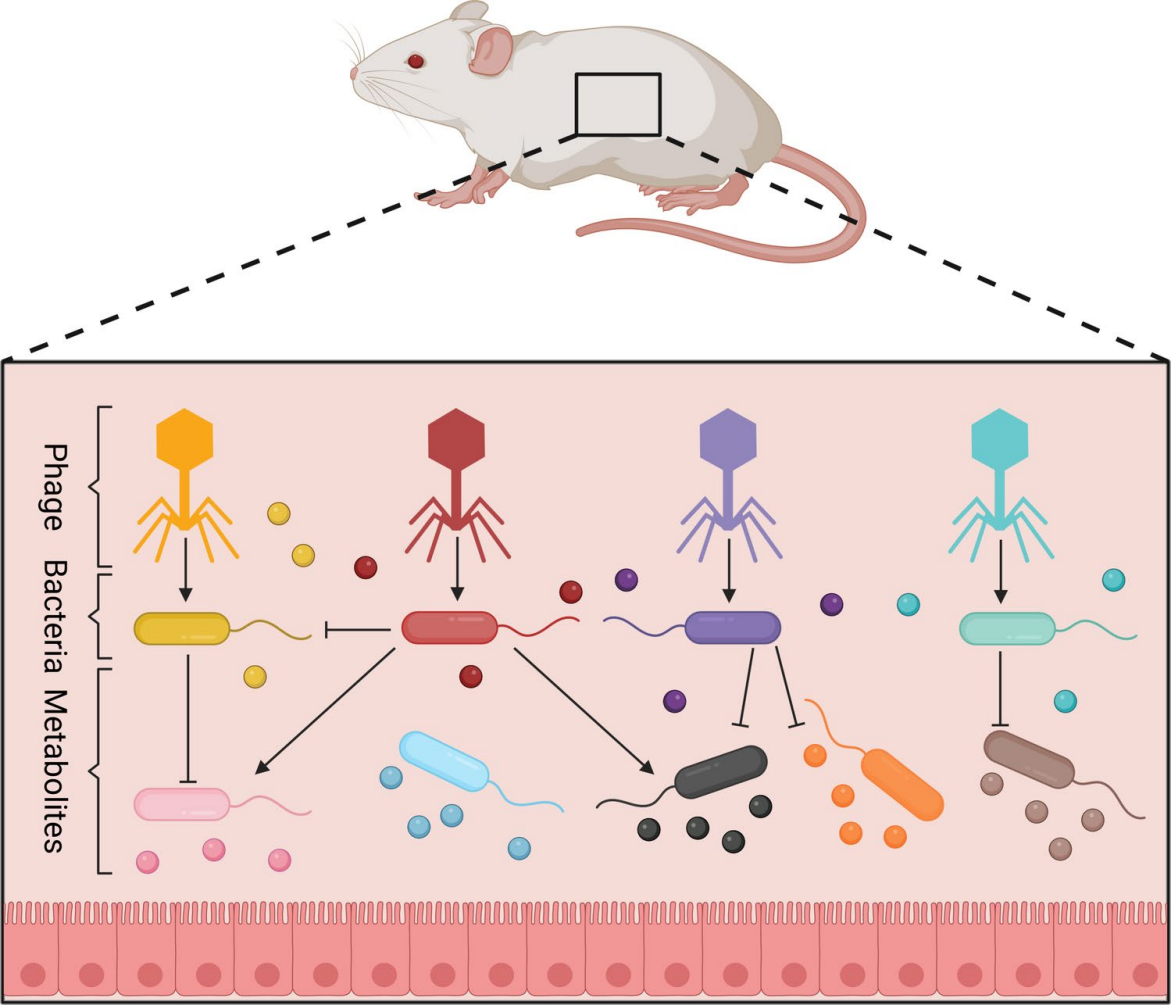
This figure illustrates the murine model’s interactions between phages, bacteria, and metabolites. The upper section shows the mouse, which is linked to the lower section, highlighting the gut’s microbial dynamics. Phages target specific bacteria, influencing bacterial behavior and metabolite production, affecting gut microbiota composition and function. The diagram demonstrates how different phages (yellow, red, purple, and cyan) interact with various bacteria, producing a range of metabolites that can potentially impact the host’s health

Phage therapy offers a targeted approach to eliminate pathogens without broad-spectrum antibiotics. In CDI, phages specific to *C. difficile* have shown promise in preclinical models. Phages can also enhance probiotic effectiveness by controlling competing bacteria (Fig. 8). However, challenges persist in culturing phages and their hosts, analyzing phage genetic diversity, and understanding their long-term effects. Technical difficulties in culturing phages and analyzing their genomes limit discovery. Single-stranded DNA and RNA phages remain poorly understood. Additionally, phages may influence bacterial responses to antibiotics, diet, and new bacterial introductions, requiring further study. FMT and bacteriophage therapy aim to restore microbial balance but differ in approach. FMT introduces entire microbial communities, while phage therapy targets specific pathogens. FMT’s success lies in its holistic restoration of diversity, but it carries risks of pathogen transmission. Phage therapy offers precision but requires a more profound understanding of phage-bacteria interactions (Lamousé-Smith et al. 2011; Liu and Forsythe 2021).

### Microbiome engineering

Microbiome engineering has emerged as a promising approach to modulating the gut microbiome, offering therapeutic potential for many disease conditions. Genetic engineering of gut microbes is a cornerstone of microbiome engineering. This involves modifying microorganisms to perform specific functions, such as producing therapeutic compounds or detecting disease biomarkers (Kim et al. 2023; Moon 2024). For instance, engineered probiotics can be designed to secrete human lysozyme, which selectively promotes beneficial microbial growth while inhibiting pathogens. Additionally, CRISPR-Cas systems have been utilized to edit the genomes of gut microbes, enabling precise modifications to enhance their therapeutic potential (Soliman and Abdellatif 2023; Wadan et al. 2024; Soliman and Mohamed 2023). Additionally, probiotics have been engineered to serve as therapeutic delivery vehicles. To target specific diseases, engineered probiotics can produce bioactive molecules, such as anti-inflammatory cytokines or enzymes. For example, probiotics engineered to express IL- 10 have shown promise in treating IBD (Patra 2024).

**Fig. 9:**
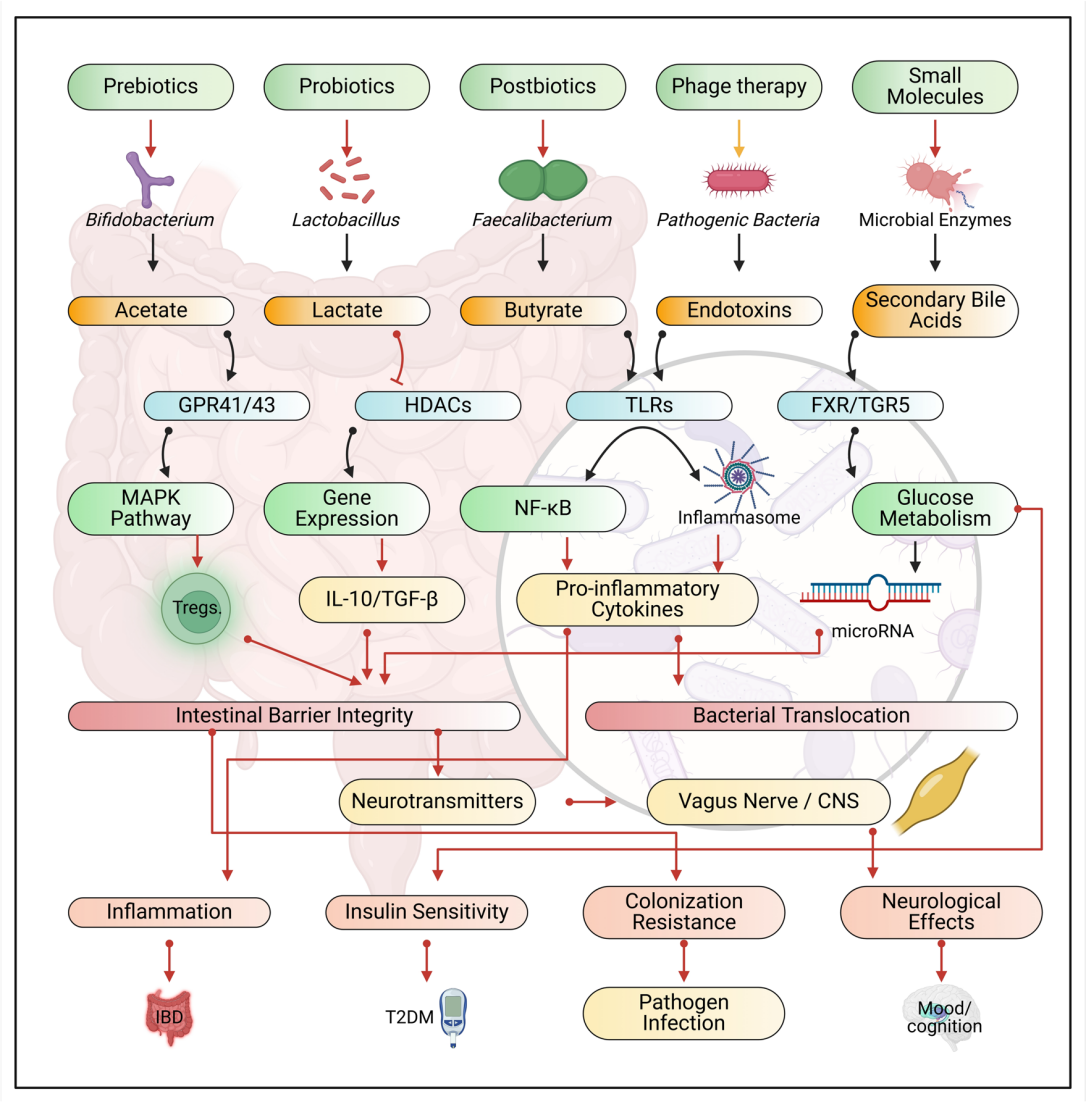
The gut microbiome diagram illustrates how prebiotics affect *Bifidobacterium* to produce acetate, which binds to GPR41/43 receptors, activating the MAPK pathway and Tregs. Probiotics involve *Lactobacillus* generating lactate that influences HDACs and gene expression, producing IL- 10/TGF-β. Postbiotics relate to *Faecalibacterium* creating butyrate, while phage therapy targets pathogenic bacteria that release endotoxins, both interacting with TLRs to trigger NF-κB and inflammasome activation, leading to pro-inflammatory cytokines. Small molecules work with microbial enzymes to produce secondary bile acids, engaging FXR/TGR5 receptors and influencing glucose metabolism connected to microRNA regulation. These molecular interactions collectively impact intestinal barrier integrity, bacterial translocation, neurotransmitter production, and vagus nerve/CNS signaling. Downstream effects manifest as inflammation (linked to IBD), altered insulin sensitivity (connected to T2DM), reduced colonization resistance against pathogen infection, and neurological effects influencing mood and cognition, demonstrating the comprehensive microbiome-gut-brain axis relationship

Recently, synthetic biology has revolutionized microbiome engineering by enabling the design of novel genetic circuits and pathways (Table 6) (Fig. 9). These advancements allow for programmable probiotics that can respond to environmental cues, such as inflammation, by releasing therapeutic molecules. Synthetic biology also facilitates the construction of microbial consortia, where multiple engineered strains work collaboratively to restore microbial balance and promote health. Engineered lysins, enzymes that lyse bacterial cells, offer a targeted approach to microbiome remodelling. These enzymes can be engineered to target harmful bacteria, restoring balance to the gut microbiome. Additionally, bacteriophage-based therapies have been explored for their ability to eliminate pathogenic bacteria while sparing beneficial microbes selectively (Rodriguez et al. 2024).

**Table 6:** Molecular pathways targeted by GMMs

Modulator class	Example compounds	Primary microbial targets	Host molecular pathways	microRNA regulation	Clinical applications	Mechanism of action
Prebiotics	Fructooligosaccharides (FOS), galactooligosaccharides (GOS), resistant starch, inulin	*Bifidobacterium* spp., *Lactobacillus* spp., *Faecalibacterium prausnitzii*	• NF-κB signaling • AMPK pathway • PPAR-γ activation • MAPK signaling • Inflammasome regulation	• miR- 155 (↓ inflammation) • miR- 146a (↑ gut barrier) • miR- 21 (↓ TLR4 signaling) • miR- 124 (↑ barrier function)	• IBD • Metabolic syndrome • Colorectal cancer prevention • Neurological disorders	• SCFA production (butyrate, propionate, acetate) • Competitive exclusion of pathogens• Enhancement of tight junction proteins
Probiotics	*L. rhamnosus* GG, *B. longum*, *L. acidophilus*, *Saccharomyces boulardii*, *Akkermansia muciniphila*	Direct supplementation	• TLR2/TLR4 signaling • JAK/STAT pathways • Wnt/β-catenin signaling • cAMP/PKA pathway • mTOR signaling	• miR- 375 (↑ insulin secretion) • miR- 29 (↓ fibrosis) • miR- 200 family (↑ epithelial integrity) • miR-Let7 (↑ anti-inflammatory action)	• Antibiotic-associated diarrhea • IBS • Hepatic encephalopathy • Atopic dermatitis	• Pathogen inhibition via bacteriocins • Immunomodulation via MAMPs • Competitive receptor binding • Bile acid metabolism
Postbiotics	Butyrate, propionate, muramyl dipeptide, lipoteichoic acid, d-alanyl-d-alanine	Direct metabolite supplementation	• GPR41/GPR43 activation • HDAC inhibition • HIF- 1α stabilization • PI3 K/Akt pathway • Nrf2 activation	• miR- 193a (↑ anti-inflammatory) • miR- 148a (↑ metabolic homeostasis) • miR- 181a (↓ pro-inflammatory) • miR- 27b (↑ barrier function)	• Ulcerative colitis • NAFLD • Obesity • Immune disorders	• Direct HDAC inhibition • G-protein coupled receptor activation • Immunomodulation • Epigenetic regulation
Phage therapy	Bacteriophage cocktails, engineered phages, CRISPR-Cas delivery phages	Pathogenic bacteria (*C. difficile*, *P. aeruginosa*, *E. coli*)	• Bacterial lysis pathways • Biofilm disruption • Quorum sensing disruption	• miR- 223 (↓ NLRP3 inflammasome) • miR- 155 (modulation of phage response)	• *C. difficile* infection • MDR bacterial infections • IBD • Precision microbiome editing	• Host-range specific lysis • Gene delivery via transduction • Biofilm disruption
Small molecule modulators	Berberine, resveratrol, curcumin, metformin, acarbose	Diverse microbial populations	• SIRT1 activation • AMPK pathway • NF-κB inhibition • mTOR signaling • PPAR signaling	• miR- 34a (↑ SIRT1) • miR- 122 (↑ metabolic homeostasis) • miR- 146a (↓ TLR4 signaling) • miR- 27a (↑ insulin sensitivity)	• T2DM • NAFLD • Cardiovascular disease • Obesity	• Quorum sensing inhibition • Enzyme inhibition • Microbial gene regulation • Selective antimicrobial activity
FMT	Processed donor stool, defined microbial consortia	Entire microbial ecosystem	• Bile acid signaling (FXR) • TLR pathways • NLRP3 inflammasome • Tryptophan metabolism • Xenobiotic metabolism	• miR- 141 (↑ intestinal homeostasis) • miR- 29 family (modulation of fibrosis) • miR- 21 (regulation of microbial recognition)	• Recurrent *C. difficile* infection • Ulcerative colitis • Metabolic syndrome • Autism spectrum disorders	• Ecosystem restoration • Competitive exclusion • Metabolic network reestablishment
Polyphenols	Quercetin, catechins, anthocyanins, ellagitannins, proanthocyanidins	*Akkermansia*, *Faecalibacterium*, *Roseburia*	• Nrf2-Keap1 pathway • MAPK signaling • NF-κB inhibition • SIRT1 activation	• miR- 155 (↓ inflammation) • miR- 16 (↑ barrier function) • miR- 27b (↑ oxidative stress response) • miR- 126 (↑ gut vascular integrity)	• IBD • Colorectal cancer • Metabolic disorders • Neuroinflammation	• Prebiotic-like effects • Antimicrobial activity • Quorum sensing inhibition • Biofilm disruption
microRNA modulators	Anti-miR oligonucleotides, miRNA mimics, extracellular vesicle delivery systems	Indirectly via host gene expression	• miR-targeted pathways • RNA interference • Epigenetic regulation • Transcriptional control	• Targeted modulation of specific miRNAs • miR-sponge technology • Bacterial miRNA cross-kingdom regulation	• IBD • Colorectal cancer • Metabolic diseases • Neurological disorders	• Modulation of host-microbe interactions • Regulation of bacterial gene expression • Control of intestinal epithelial cell function
Bacteriocins and antimicrobial peptides	Nisin, microcin, LL- 37, defensins, bacteriocin-producing probiotics	Specific bacterial taxa	• Pore formation pathways • Cell wall synthesis inhibition • Disruption of membrane potential	• miR- 152 (↑ antimicrobial peptide production) • miR- 23a (regulation of defensin expression)	• *C. difficile* infection • SIBO • Periodontal disease • Targeted pathogen reduction	• Selective antimicrobial activity • Pore formation • Inhibition of cell wall synthesis • Disruption of membrane potential
Artificially engineered bacteria	SYNB1618 (PKU), SYNB1934, SYNB8802	Engineered strains of *E. coli* Nissle 1917, *Lactococcus lactis*	• Phenylalanine metabolism • Ammonia detoxification • GABA production • IL- 10 delivery • GLP- 1 secretion	• Designed microRNA production and delivery • Circuit-controlled miRNA expression	• Phenylketonuria • Hyperammonemia • IBD • T1DM • Obesity	• Engineered metabolic pathways • Therapeutic protein secretion • Regulated gene circuits • Environmental sensing
Gut-brain axis modulators	5-HTP, GABA, serotonin precursors, psychobiotics	*Lactobacillus* and *Bifidobacterium* with neuroactive capacity	• Vagus nerve signaling • HPA axis • Serotonin-tryptophan pathway • Kynurenine pathway • BDNF signaling	• miR- 132 (↑ neuroplasticity) • miR- 124 (↑ neuronal differentiation) • miR- 29 family (↓ neuroinflammation) • miR- 206 (BDNF regulation)	• Depression • Anxiety • Autism • Parkinson’s disease • Alzheimer’s disease	• Neurotransmitter production • Vagal afferent stimulation • Microglia modulation • Neuroinflammation reduction
Extracellular vesicles (EVs)	Bacterial EVs, probiotic-derived EVs, engineered EVs	Multiple bacterial species	• Intracellular signaling • JAK/STAT pathways • Endocytosis pathways • TLR signaling • Inflammasome regulation	• EV-delivered microRNAs • miR- 30 d (immunomodulation)• miR- 148a (metabolic regulation) • miR- 155 (inflammation control)	• IBD • Autoimmune disorders • Cancer immunotherapy • Metabolic disorders	• Lipid transfer • Protein delivery • Nucleic acid transport • MAMPs presentation

Microbiome engineering also aims to rewire host-microbiome interactions. Engineered bacteria can influence host metabolism, immune function, and neurological processes (Mousavinasab et al. 2023). For example, engineered bacteria that produce neurotransmitter precursors have been investigated for their potential to modulate mental health conditions. Microbiome engineering has shown promise in treating metabolic diseases like obesity and type 2 diabetes. Engineered microbes can regulate glucose metabolism and improve insulin sensitivity, offering a novel approach to managing these conditions (Barra et al. 2020; Meng et al. 2024). Additionally, IBD and other immune-mediated disorders have been targeted through microbiome engineering. Engineered probiotics that deliver anti-inflammatory molecules directly to the gut have effectively reduced inflammation and restored epithelial barrier function (Rutter et al. 2022; Shen et al. 2022). The gut microbiome’s role in cancer progression and treatment response has led to the exploration of microbiome engineering in oncology. Engineered bacteria can deliver anticancer drugs directly to tumors, minimizing systemic toxicity and enhancing therapeutic efficacy (Porcari et al. 2024).

The safety of engineered microbes is a critical consideration. Potential risks include unintended off-target effects and the possibility of engineered microbes persisting in the environment. Strategies such as genetic kill switches and biocontainment mechanisms are being developed to mitigate these risks (Moon et al. 2024; Lee et al. 2023). Also, the regulatory landscape for microbiome engineering is still evolving. Ensuring the safety and efficacy of engineered microbial products while addressing ethical concerns, such as potential misuse, is essential for advancing this field (Zhang et al. 2021b).

## Personalized GMM therapies: a frontier in precision medicine

Personalized GMM therapies, tailored to an individual’s microbiota profile, represent a groundbreaking advancement in precision medicine. Using metagenomic sequencing, clinicians can analyze a patient’s gut microbiome to detect microbial imbalances or dysbiosis, enabling the development of targeted GMM interventions (Dinan et al. 2019). This approach allows for precise modifications to restore microbial equilibrium, offering customized treatment strategies. For instance, in inflammatory bowel disease (IBD), metagenomic analysis may reveal a deficiency in anti-inflammatory bacteria such as *Faecalibacterium prausnitzii*. In such cases, GMMs could be engineered to supplement these bacteria or produce metabolites like butyrate, which enhance gut barrier function and reduce inflammation (Strandwitz et al. 2019). Beyond gastrointestinal diseases, personalized GMM therapies hold significant potential in optimizing treatment across various conditions. In cancer, the gut microbiome plays a crucial role in modulating immunotherapy efficacy. By identifying microbial signatures linked to poor treatment response, clinicians can administer GMMs designed to enhance the gut microbiota, thereby improving the effectiveness of immune checkpoint inhibitors (Aggarwal et al. 2020b). Similarly, in neuropsychiatric disorders such as depression and anxiety, where the gut-brain axis is a key regulator, GMMs could be tailored to produce neurotransmitters like GABA or serotonin precursors, aligning with the patient’s specific microbial and metabolic profile (Canale et al. 2021).

Personalized GMM therapies also offer preventative potential. Individuals at high risk of metabolic disorders, such as type 2 diabetes, could benefit from engineered GMMs designed to produce metabolic regulators like glucagon-like peptide- 1 (GLP- 1) based on their unique microbiome composition, potentially delaying disease onset and improving long-term health outcomes (Gerber et al. 2018). Despite these promising applications, challenges remain in implementing personalized GMM therapies. The complexity and cost of metagenomic sequencing and data interpretation may limit accessibility, particularly in low-resource settings (Hwang et al. 2017). Additionally, the dynamic nature of the gut microbiota means that therapies may require periodic adjustments to maintain efficacy. Nonetheless, as research advances and technology becomes more accessible, personalized GMM therapies have the potential to transform precision medicine by providing highly individualized treatments that address the root causes of disease, paving the way for a more tailored and practical approach to healthcare (Mimee et al. 2016).

## The economic impact of GMMs’ repurposing

The repurposing of GMMs for therapeutic applications carries significant economic implications, offering a cost-effective and efficient alternative to traditional drug development. By leveraging well-characterized probiotic strains such as *Lactobacillus* and *Bifidobacterium*, researchers can bypass some of the most expensive and time-consuming stages of drug development, such as extensive safety trials, allowing for a faster transition from research to clinical application (Moon et al. 2024). This streamlined approach is particularly beneficial for addressing unmet medical needs in areas such as cancer, inflammatory diseases, and metabolic disorders, where conventional treatments are often expensive or ineffective (O’Toole et al. 2017; Riglar and Silver 2018).

The expanding global probiotics market, projected to surpass $3 billion by 2024, reflects the increasing demand for microbial-based interventions in pharmaceuticals, dietary supplements, and functional foods (Aggarwal et al. 2020c). Advances in synthetic biology and microbiome research have further driven this growth by expanding the potential applications of GMMs beyond traditional uses, including their role in targeted drug delivery for conditions such as IBD, neurological disorders, and cancer. However, despite the economic potential of GMM-based therapies, challenges remain in ensuring equitable access (Cryan et al. 2019). The high costs of research, development, and manufacturing, along with intellectual property restrictions, could limit affordability in low-income settings, exacerbating health disparities (Moon et al. 2024). For instance, while GMM-based treatments for IBD or cancer may be highly effective, their availability may be restricted to wealthier populations unless policies are implemented to promote accessibility (O’Toole et al. 2017). Addressing these challenges requires strategic public–private partnerships (PPPs) that unite academic institutions, biotechnology companies, and government agencies to support the development and distribution of affordable GMM therapies. Government funding can facilitate early-stage research, while private companies can contribute expertise in large-scale production and distribution (Zhao et al. 2020b). Additionally, non-profit organizations can advocate for pricing models and subsidy programs to ensure GMM-based treatments reach underserved populations, particularly in low- and middle-income countries (LMICs), where the burden of disease is highest (Aggarwal et al. 2020c).

Beyond market expansion and accessibility, the integration of GMM-based therapies into healthcare systems could yield substantial economic benefits by reducing the overall cost of disease management. For example, targeted microbial therapies for chronic conditions such as IBD and diabetes could minimize the need for expensive surgeries, hospitalizations, and long-term medication use, ultimately lowering healthcare expenditures. By addressing disease mechanisms at the microbial level, GMMs have the potential to enhance patient outcomes while alleviating the economic burden of complications and comorbidities, creating a more sustainable and cost-efficient healthcare system (Cryan et al. 2019).

## Regulatory hurdles in the use of GMMs

Regulatory challenges must be addressed to ensure the safe and effective implementation of GMMs in therapeutic applications (Gopalakrishnan et al. 2018; Mimee et al. 2016). These challenges involve safety, ethics, and standardization, requiring collaboration among researchers, regulators, and industry stakeholders to establish clear guidelines. Regulatory agencies such as the FDA and EMA mandate extensive testing to assess toxicity, colonization, and potential off-target effects before clinical trials can confirm therapeutic outcomes (Strandwitz et al. 2019; Abbas et al. 2022b). For instance, GMMs engineered to produce anti-inflammatory cytokines or deliver chemotherapeutic agents must undergo rigorous evaluation to prevent unintended immune responses or microbiota disruptions, but these lengthy and costly testing processes pose significant barriers to commercialization (Aggarwal et al. 2020c).

Beyond safety concerns, ethical issues arise regarding gene transfer risks to native microbiota or the environment, potentially leading to unforeseen ecological or health impacts (Kassam et al. 2017). The long-term effects of introducing GMMs into the human body remain unclear, necessitating continuous research and transparent communication with the public to monitor their implications (Parvathaneni et al. 2019b). Additionally, the lack of standardized protocols for GMM production and administration complicates regulatory approval, as variations in manufacturing, storage, and delivery methods can affect viability and therapeutic consistency. Standardization efforts are essential to ensure reproducibility and safety, requiring coordinated efforts between researchers, manufacturers, and regulators (Brodmann et al. 2017).

Addressing these regulatory challenges will require collaborative efforts to streamline approval processes while maintaining rigorous safety standards (de Vos et al. 2022; Kurtz et al. 2019). Clearer guidelines from regulatory agencies can facilitate the development of GMM-based therapies, while cooperation between researchers and manufacturers can establish standardized protocols for production and administration (Sanders et al. 2019; van Loo et al. 2020). Public–private partnerships can also help address ethical concerns and improve accessibility, ensuring that GMM-based treatments are available to all patients regardless of socioeconomic status (Veiga et al. 2020). By overcoming these hurdles, GMMs can be widely adopted, paving the way for innovative and targeted therapies for various diseases.

## Conclusion and future perspectives

This review investigates the feasibility of repurposing GMMs for the management of various disorders and delves into an array of interventions aimed at gut microbiota modulation. These interventions encompass dietary modifications, fecal microbiota transplantation, and the application of bacteriophages, microbiome engineering, and the modulation of the immune system. Integrating pharmacological and non-pharmacological strategies in modulating the gut microbiome presents a promising frontier in enhancing health outcomes and therapeutic efficacy. Pharmacological repurposing of existing drugs alongside the application of genetically modified microorganisms can revolutionize treatment options, reduce developmental timelines, and improve accessibility to essential therapies. The projected growth of the probiotics market further underscores the increasing consumer demand for GMM-related health benefits and their critical role in strengthening immune responses and optimizing vaccine effectiveness. On the non-pharmacological side, dietary modulation, FMT, exercise, sleep hygiene, stress management, and surgical options like gastric bypass are crucial for maintaining a balanced microbiome and promoting overall health. The evidence supporting these strategies highlights their ability to stimulate beneficial microbial growth, combat pathogenic species, and improve gut function across diverse populations. As the understanding of microbiome dynamics continues to evolve, future research should focus on optimizing these interventions in clinical practice and exploring their combined effects. The synergistic potential of pharmacological and non-pharmacological approaches offers a holistic framework for addressing a wide array of health issues, making it imperative for future studies to delve deeper into the interplay between gut microbiota and overall human health. By leveraging both methodologies, we can pave the way for more effective, individualized health solutions that ultimately improve quality of life.

## Data Availability

All source data for this work (or generated in this study) are available upon reasonable request.

## Abbreviations

*AMPK*: AMP-activated protein kinase
*FXR*: Farnesoid X receptor
*GLP- 1*: Glucagon-like peptide-1
*HDAC*: Histone deacetylase
*HPA*: Hypothalamic-pituitary-adrenal
*IBS*: Irritable bowel syndrome
*MAPK*: Mitogen-activated protein kinase
*MDR*: Multi-drug resistant
*NAFLD*: Non-alcoholic fatty liver disease
*NLRP3*: NOD-, LRR-, and pyrin domain-containing protein 3
*PI3 K*: Phosphoinositide 3-kinase
*PKU*: Phenylketonuria
*SCFA*: Short-chain fatty acid
*SIRT1*: Sirtuin 1
*T2DM*: Type 2 diabetes mellitus
*TLR*: Toll-like receptor
*BDNF*: Brain-derived neurotrophic factor
*GABA*: Gamma-aminobutyric acid
*GPR*: G protein-coupled receptor
*HIF*: Hypoxia-inducible factor
*IBD*: Inflammatory bowel disease
*JAK/STAT*: Janus kinase/signal transducer and activator of transcription
*MAMPs*: Microbe-associated molecular patterns
*mTOR*: Mammalian target of rapamycin
*NF-κB*: Nuclear factor kappa-light-chain-enhancer of activated B cells
*Nrf2*: Nuclear factor erythroid 2-related factor 2
*PKA*: Protein kinase A
*PPAR*: Peroxisome proliferator-activated receptor
*SIBO*: Small intestinal bacterial overgrowth
*T1DM*: Type 1 diabetes mellitus
*5-HTP*: 5-Hydroxytryptophan
